# Association of educational attainment with esophageal cancer, Barrett's esophagus, and gastroesophageal reflux disease, and the mediating role of modifiable risk factors: A Mendelian randomization study

**DOI:** 10.3389/fpubh.2023.1022367

**Published:** 2023-03-28

**Authors:** Xuening Zhang, Xiaorong Yang, Tongchao Zhang, Xiaolin Yin, Jinyu Man, Ming Lu

**Affiliations:** ^1^Department of Epidemiology and Health Statistics, School of Public Health, Cheeloo College of Medicine, Shandong University, Jinan, China; ^2^Clinical Epidemiology Unit, Qilu Hospital of Shandong University, Jinan, China; ^3^Clinical Research Center, Qilu Hospital of Shandong University, Jinan, China

**Keywords:** education, esophageal cancer, Barrett's esophagus, gastroesophageal reflux disease, Mendelian randomization, mediation analysis

## Abstract

**Background:**

Observational studies have reported that educational attainment has been related to the risk of esophageal cancer (EC) and its precancerous lesions. However, the causal relationship remains controversial. We aimed to apply the Mendelian randomization (MR) design to determine the causal associations between genetically predicted educational attainment and EC, Barrett's esophagus (BE), and gastroesophageal reflux disease (GERD), and to explore whether modifiable risk factors play a mediating role.

**Methods:**

Using summary statistics from genome-wide association studies (GWASs) based on European ancestry individuals of several years in education (EduYears, primary analysis, *n* = 293,723), college completion (College, secondary analysis, *n* = 95,427), EC (*n* = 420,531), BE (*n* = 361,194), and GERD (*n* = 420,531), genetic associations between two education phenotypes and EC, BE, and GERD were tested by two-sample MR analyses. Then, two-step MR mediation analyses were used to assess the proportion of the aforementioned association that might be mediated by body mass index (BMI), major depressive disorder (MDD), smoking, drinking, carbohydrates, fat, and protein intake.

**Results:**

Genetically predicted EduYears was negatively associated with the risk of EC, BE, and GERD {odds ratio (OR), 0.64 [95% confidence interval (CI) 0.44–0.94], 0.86 (95% CI, 0.75–0.99), and 0.62 (95%CI, 0.58–0.67)}. EduYears was negatively associated with BMI, MDD, and smoking (range of OR: 0.76–0.84). There were positive associations between BMI, smoking with EC, BE, and GERD, as well as between MDD with GERD (range of OR: 1.08–1.50). For individual mediating effect, BMI and smoking mediated 15.75 and 14.15% of the EduYears-EC association and 15.46 and 16.85% of the EduYears-BE association. BMI, MDD, and smoking mediated 5.23, 4.98, and 4.49% of the EduYears-GERD association. For combined mediation, the aforementioned mediators explained 26.62, 28.38, and 11.48% of the effect of EduYears on EC, BE, and GERD. The mediating effects of drinking and dietary composition were not significant in the effect of education on EC, BE, and GERD.

**Conclusion:**

Our study supports that genetically predicted higher educational attainment has a protective effect on EC, BE, and GERD, and is partly mediated by reducing adiposity, smoking, and depression.

## 1. Introduction

Esophageal cancer (EC) is the eighth most common malignancy and the sixth leading cause of cancer-related deaths worldwide, with an estimated 604,100 new cases and 544,076 deaths in 2020 (1). Esophageal adenocarcinoma (EAC) develops rapidly and becomes the predominant subtype of EC in Caucasian populations from Europe, North America, and Australia (2). Barrett's esophagus (BE) is a common precursor lesion to EAC. This precancerous lesion is caused by recurrent esophageal cell proliferation resulting from long-term gastroesophageal reflux disease (GERD) (3). The primary prevention of cancer can be realized through studying the same influencing factors of EC and its precancerous lesions and disease and then giving sufficient attention and improvement measures. Previous observational studies have suggested that educational attainment was associated not only with EC but also with its precancerous lesions and disease including BE and GERD (4–7). Compared with other socioeconomic factors that may change over time, such as occupation and income, educational attainment is largely determined early in life and comparatively easy to measure (8). Therefore, educational attainment can be used as a good proxy of socioeconomic position to explore the causal associations with EC, BE, and GERD risk.

Previous observational mediation studies showed that modifiable risk factors partially mediated the association between education and other diseases (9, 10). EC, BE, and GERD have been identified to be associated with metabolic (11), psychological (12, 13), lifestyle (14, 15), and dietary factors (16) in observational studies. However, whether and to what extent these modifiable risk factors explain the total effect of education on EC, BE, and GERD has not been investigated. From the perspective of disease prevention, improving educational attainment among the population requires intervention early in life, which is beyond the scope of most clinical practices. In contrast, mediators of lifestyle behaviors may be more susceptible to intervention. Therefore, estimating the mediated effect of modifiable risk factors for the association between educational attainment and EC and its precancerous lesions has broad implications for our understanding of the causes of EC and the potential development of prevention approaches.

Due to the methodological limitations of traditional observational research, such as uncertain temporal relationships, insufficient sample sizes, short follow-up periods, or potential confounding factors, the foregoing associations may not stem from an underlying causal effect. In addition, given the long interval between education and EC, randomized controlled trials (RCTs) are difficult to implement in this field. Therefore, it is necessary to improve causal inference through other study designs. Mendelian randomization (MR) uses randomly assigned genetic variants [single-nucleotide polymorphisms (SNPs)] during conception as instrumental variables for exposure, which could minimize measurement errors, confounding, and reverse causality (17). The public availability of large-scale genome-wide association studies (GWASs) data provides an opportunity to perform two-sample MR analysis using summary statistics from multiple sources, which substantially increases the statistical power. The extension of MR methodology, including multivariable MR (MVMR) and two-step MR, leads to the possibility to explore mediators between exposure and outcome while avoiding unmeasured confounding (18).

In this study, we aimed to access whether there is a potential causal association between educational attainment and EC, BE, and GERD. In addition, we aimed to access whether modifiable risk factors mediate the effect of educational attainment on EC, BE, and GERD.

## 2. Materials and methods

### 2.1. Study design

This study used summary data published by multiple GWAS; ethical approval and patient consent were obtained by corresponding studies (19–26). This study was reported according to the Strengthening the Reporting of Observational Studies in Epidemiology Using Mendelian Randomization (STROBE-MR) checklist (17).

**Figure 1:**
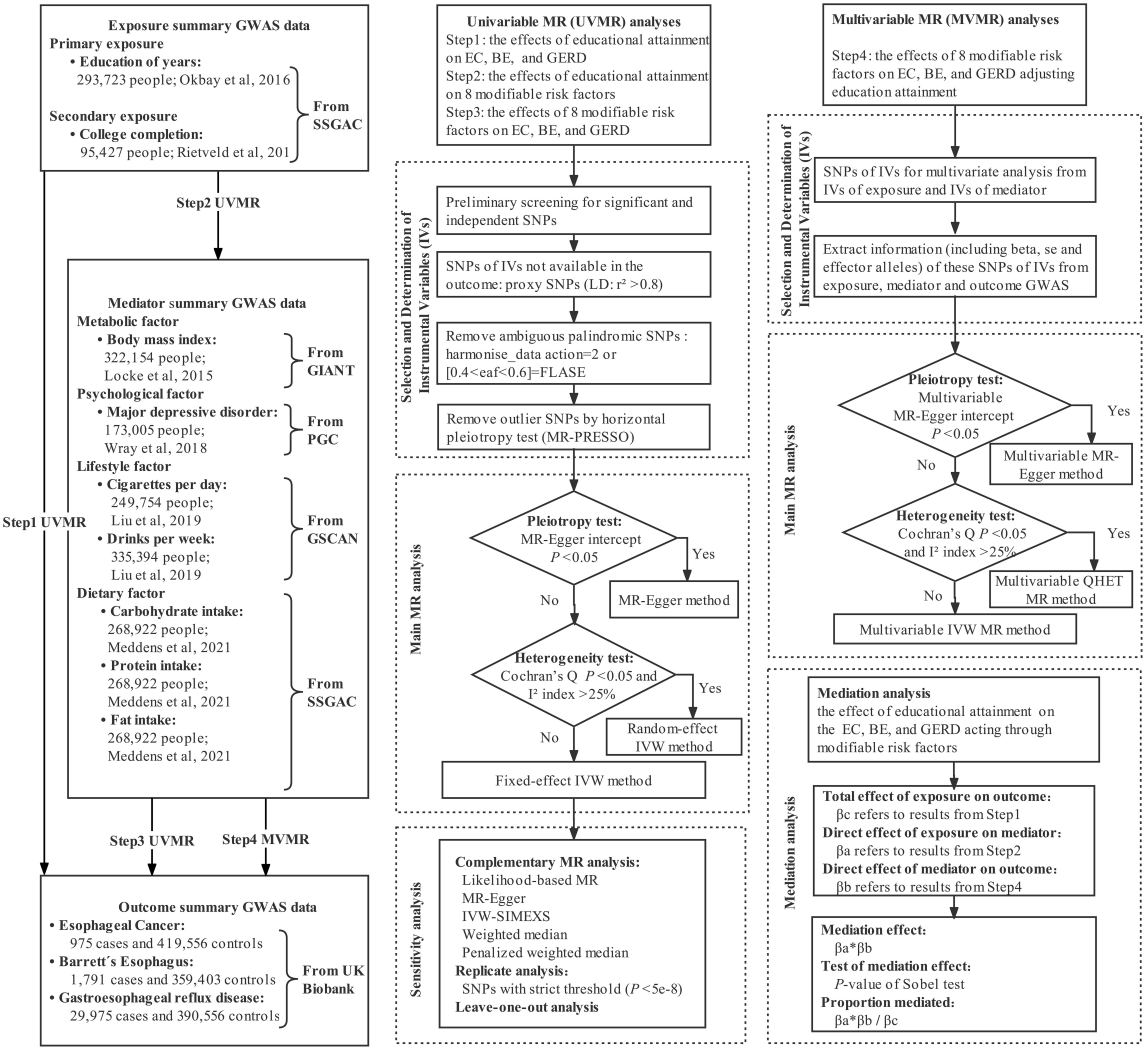
Flowchart of MR analyses in this study.

We performed univariable, multivariable, and two-step two-sample MR mediation analyses to investigate whether genetically predicted educational attainment was causally associated with the risk of EC, BE, and GERD, and to assess the proportion mediated by modifiable risk factors in the aforementioned associations. The flowchart of MR analysis in this study is shown in Figure 1.

### 2.2. GWAS data for exposure

For educational attainment, we used two exposure phenotypes including the number of years in education (EduYears, primary analyses) (19) and college completion (College, secondary analyses) (20). At present, the GWAS for EduYears from the 2018 study by Lee et al. was widely used. However, this GWAS included UK Biobank individuals, which resulted in sample overlap between exposure and outcome (21). To avoid sample overlap bias in two-sample MR, we selected the largest GWAS excluding UK Biobank individuals as the primary analysis, which was widely used in the causal correlation analysis between education and other outcomes (27–32). GWAS from Lee et al. was used in the sensitivity analysis. Genetic association results for EduYears and College were identified from the Social Science Genetic Association Consortium (SSGAC) GWAS meta-analysis of 293,723 and 95,427 European individuals, excluding individuals from the UK Biobank study. EduYears was a quantitative variable, and its average is 14.3 [standard deviation (SD) = 3.6]. College was a binary variable and was defined as Yes/No completion of university education. A detailed description of participating cohorts and GWAS is shown in Supplementary Table S1, Supplementary eMethod 1.

### 2.3. GWAS data for mediator

The mediators represented four categories of modifiable risk factors, which contain a lot of variables. Thus, we selected several variables which have been widely reported to be associated with esophageal disorders found in observational studies, which were metabolic factor [i.e., body mass index (BMI)], psychological factor [i.e., major depressive disorder (MDD)], lifestyle factor (i.e., smoking quantity and alcohol consumption), and dietary factor (i.e., carbohydrate, protein, and fat intake). Genetic association results for BMI were extracted from GWAS meta-analysis published by the Genetic Investigation of ANthropometric Traits (GIANT) Consortium, totaling 322,154 individuals of European ancestry (22). Genetic association estimates for MDD were derived from the GWAS meta-analysis published by Psychiatric Genomics Consortium (PGC) based on 173,005 European ancestries (59,851 MDD cases and 113,154 controls) from the PGC29 cohort and the five additional cohorts (23). Genetic association results for smoking quantity and alcohol consumption were derived from GWAS meta-analysis conducted by the GWAS and Sequencing Consortium of Alcohol and Nicotine use (GSCAN) consortium (24). Cigarettes per day (smoking, *N* = 249,752) is defined as the average number of cigarettes smoked per day by current or former smokers. Drinks per week (drinking, *N* = 335,394) is defined as the average number of all types of alcohol consumed per week, which reflected common alcohol drinking behavior, not excessive or harmful drinking behavior. For dietary factors, we utilized the GWAS meta-analysis of European ancestry individuals (carbohydrate/fat/protein: *N* = 268,922) (25).

### 2.4. GWAS data for outcome

Summary-level genetic data for EC, BE, and GERD was obtained from the UK Biobank which was a population-based cohort study that recruited more than 500,000 volunteers aged 40–69 years between 2006 and 2010. We used the second-round analysis of UK Biobank data from the Pan-ancestry genetic analysis of the UK Biobank (Pan-UK Biobank). In this project, participants have been divided into six ancestry groups to account for population stratification, and we only restricted the analysis to individuals of European ancestry to avoid bias caused by population stratification. EC (975 cases and 419,556 controls), BE (1,791 cases and 359,403 controls), and GERD (29,975 cases and 390,556 controls) with European ancestry were extracted from the Pan-UK Biobank website and IEU OpenGWAS project website (26). All disease diagnoses were based on the national registries (International Classification of Diseases, 10th revision code). The variable definitions and data collection for exposure, mediator, and outcome GWAS are described in detail in Supplementary eMethod 1.

### 2.5. Statistical analysis

The two-sample MR is an approach that uses a set of SNPs as instrumental variables (IVs) to estimate the causal effect of exposure or mediator on the outcome, where the valid IVs need to satisfy three core assumptions: (1) the relevance assumption, instrumental variables were associated with the exposure, (2) the independence assumption, instrumental variables were not associated with any confounders of the exposure-outcome association, (3) and the exclusion restriction assumption, instrumental variables were only associated with the outcome through the exposure and potential mediators (33).

#### 2.5.1. Selection of genetic instrumental variables for MR analysis

The SNPs of genetic IVs were screened and determined by the following steps. First, GWAS were obtained for each exposure and mediator, and SNPs associated with each trait at genome-wide significance (*P* < 5e-8) were identified. Independent SNPs loci were obtained by clumping with a threshold of linkage disequilibrium (LD) *r*^2^ < 0.001 and a distance of 10,000 kb. Since the explained variance (*R*^2^) of almost all IVs to exposure under a strict criterion was <1%, SNPs using a more relaxed statistical threshold (*P* < 5e-5) were extracted to improve the explained variance of IVs. This relaxed statistical threshold of the genetic IVs approach had been described in previous MR studies (34, 35). Second, SNPs for IVs of exposure and mediator were extracted from GWAS data of outcome. If SNPs for IVs were absent in the summary GWAS statistics, the LDlink tool (36) was used to identify proxy SNPs of European ancestry using high LD with *r*^2^ > 0.8. Third, the direction of effects between exposure and outcome associations were harmonized, that was to align the variants to the effect allele. Palindromic SNPs were inferred based on the allele frequency (AF) by setting action = 2 (If the AF information of palindromic SNPs was incomplete, the SNPs with AF between 0.4 and 0.6 were regarded as ambiguous palindromes and removed) (37). Finally, one of the key hypotheses of MR is the instrumental variables only act on the outcome *via* the exposure and/or potential mediators. The violation of this assumption is known as horizontal pleiotropy. MR analyses are needed to exclude pleiotropic instrumental variables that are associated with the outcome through pathways other than the exposure/mediators. Such SNPs were defined as those with a genome-wide significant association with alternative pathways in GWAS conducted by representative consortiums, as revealed by the PhenoScanner website (38). If MR pleiotropy residual sum and outlier (MR-PRESSO) identified outliers, the MR analyses were conducted after the exclusion of the outliers (39). The final genetic IVs were obtained through the aforementioned screening process. F-statistic for each SNP of more than 10 indicated that weak instruments had a relatively low-risk bias (40).

#### 2.5.2. Univariable MR analysis

The primary method for univariate MR analysis was determined based on pleiotropy and heterogeneity. First, the MR-Egger intercept test was conducted to evaluate whether there was the presence of potential pleiotropy (41). If there was significant horizontal pleiotropy, the MR-Egger regression was used; otherwise, the inverse-variance weighted (IVW) method was used (42, 43). IVW method assumes that all SNPs are valid instruments or are invalid in such a way that the overall bias is zero, which has been described in detail in the previous study (44). Notably, the existence of significant horizontal pleiotropy was extremely rare due to the rigorous screening of instrumental variables. Then, Cochran's *Q* statistic and *I*^2^ index were used to test for the presence of heterogeneity (*P* < 0.05 and *I*^2^ > 25% were considered statistically significant) (45). If Cochran's *Q* and *I*^2^ index indicated potential heterogeneity, the random-effect IVW model was used; otherwise, the fixed-effect IVW model was used. As many MR analyses with multi-exposures and multi-outcomes did, to preserve the type I error of the global null hypothesis of all tested associations being in fact null, multiple testing was required. In addition, we note that these outcomes are related to each other, i.e., the tests are not completely independent of each other, and the Bonferroni correction may be conservative. Therefore, we used the Benjamini–Hochberg method to control the false discovery rate (FDR), with *q* < 0.05 for FDR as significant evidence of associations, and between *q* > 0.05 for FDR and uncorrected *P* < 0.05 as suggestive evidence of associations (46). This method has been widely used in previous studies (47).

#### 2.5.3. Multivariable MR analysis

First, SNPs of IVs for multivariate MR analysis were derived from the combination of SNPs of IVs for each exposure and mediator in univariable MR analysis. Second, we extracted information (including beta, se, and effect alleles) of these SNPs of IVs from GWASs of exposure, mediator, and outcome, and harmonized effect allele. Third, a multivariable MR-Egger intercept test was performed to test for potential pleiotropy. If there was significant pleiotropy, a multivariable MR-Egger method was used for MR analysis (48). When there was no significant pleiotropy, *I*^2^ and Cochran's *Q* were used to further assess whether there was heterogeneity. If there was significant heterogeneity, the multivariable QHET method was used; otherwise, the multivariable IVW method was used for MR analysis (49).

#### 2.5.4. Mediation analysis

**Figure 2:**
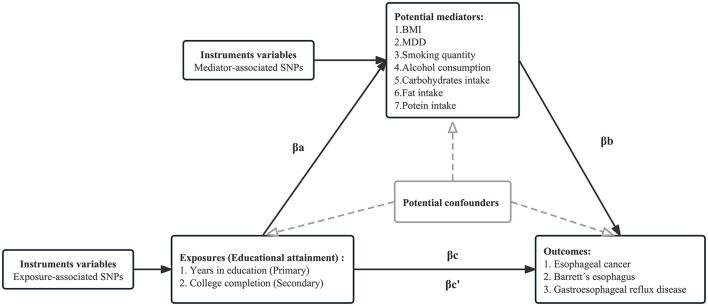
Directed acyclic graph of the MR mediation analysis. The total effect of exposure on the outcome, βc, was derived using univariable MR (i.e., genetically predicted EduYears as exposure and EC as outcome). The total effect was decomposed into (i) indirect effect (mediation effect) using a two-step approach (where βa is the effect of EduYears on BMI, and βb is the effect of BMI on EC adjusting for EduYears) and the product method (βa * βb) and (ii) direct effect (βc′ = βc – βa*βb). The same process is applied to obtain the individual mediation effect of each mediator. We divided the individual mediated effect by the total effect to estimate the individual proportion mediated. The combined mediation effect is calculated by accumulating each mediation effect under multivariable MR of multiple parallel mediation analyses. We divided the combined mediated effect by the total effect to estimate the total proportion mediated.

The directed acyclic graph of the MR mediation analysis is shown in Figure 2. The process of two-step MR analysis is as follows. First, univariable MR (UVMR) was used to approach estimated the effect of each exposure on each mediator. Second, regression-based multivariable MR (MVMR) was applied to estimate the effect of each mediator on the risk of the outcome, adjusting for exposure (50). Third, the individual mediation effect (or indirect effect) of each mediator was calculated by multiplying the effect of each exposure on each mediator with the effect of each mediator on the outcome adjusted exposure (51). To obtain unbiased estimates of the direct and indirect effects, MR mediation analysis assumed that there is no interaction between the exposure and mediator, and there is a linear association between the exposure, mediator, and outcome (18). Finally, we divided the mediation effect by the total effect to estimate the proportion mediated. The calculation of standard error is based on the delta method. MVMR of multiple parallel mediation analysis was used to adjust the genetic effect of several mediators simultaneously to estimate the combined mediation effect. Detailed methods are presented in Supplementary eMethod 2.

#### 2.5.5. Sensitivity analysis

A series of sensitivity analyses were performed to ensure the robustness of our findings. First, complementary MR analysis methods [including likelihood-based MR (52), MR-Egger (53), IVW-SIMEX (simulation extrapolation) (54), weighted median, and penalized weighted median (55)] with different assumptions were carried out to help verify the causal estimate when there was a few invalid IVs or horizontal pleiotropy bias. Consistency of effect direction across methods indicated a more reliable causality inference. Second, in order to correct the potential weak IVs bias caused by relaxing the threshold (*P* < 5e-5) and improve statistical efficiency and estimation accuracy of models, we applied the Radial IVW method (56) and re-performed MR analysis under strict threshold (*P* < 5e-8). Third, to verify the representativeness of the IVs of EduYears in the main analysis, the MR analysis was repeated using education with the largest GWAS whereas with a large overlap with the outcome sample. Finally, a leave-one-out sensitivity analysis was performed by eliminating a single SNP from the MR analysis one by one to assess the effect of individual variation on causal estimation.

All statistical analyses were performed using R version 4.1.0 and based on the TwoSampleMR (version 0.5.6) R package.

## 3. Results

### 3.1. Selection of genetic instrumental variables for each MR analysis

The GWAS data sources of each trait are shown in Table 1. The final IVs of EduYears trait for modifiable risk factors, EC, BE and GERD explained about 3.12% of the variance (*F*-statistic: 24.6–25.1). The final IVs of modifiable risk factors for EC, BE, and GERD explained about 1.13–2.70% of the variance (*F*-statistic: 16.6–32.9) (Supplementary Tables S2–S4).

**Table 1:** GWAS data sources and information included in the current study.

**Trait**	**Phenotype**	**Unit**	**Sample size**	**Ancestry**	**Adjustments**	**GWAS data source**	**PubMed ID**
Educational attainment	Number of years in education	SD increase in years of education	293,723	European	Age, sex, and the first 10 principal components	Okbay et al. (19)	27225129
	College completion	Log-odds of college completion	95,427	European	Age, sex, and first four principal components	Rietveld et al. (20)	23722424
Metabolic factor	Body mass index	SD increase in Body mass index	322,154	European	Age, age, and study-specific covariates (e.g., genotype-derived principal components)	Locke et al. (22)	25673413
Psychological factor	Major depressive disorder	Log-odds of Major depressive disorder	173,005	European	Age, sex, and principal components	Wray et al. (23)	29700475
Lifestyle factor	Cigarettes smoked per day	SD increase in cigarettes smoked per day	249,754	European	Age, sex and the first 10 genetic principal components	Liu et al. (24)	30643251
	Drinks per week	SD increase in drinks per week	335,394	European	Age, sex and the first 10 genetic principal components	Liu et al. (24)	30643251
Dietary factor	Carbohydrate intake	SD increase in carbohydrate intake	268,922	European	Age, sex, and the first 10 principal components	Meddens et al. (25)	32393786
	Fat intake	SD increase in fat intake	268,922	European	Age, sex, and the first 10 principal components	Meddens et al. (25)	32393786
	Protein intake	SD increase in protein intake	268,922	European	Age, sex, and the first 10 principal components	Meddens et al. (25)	32393786
Esophageal diseases	Esophageal cancer	-	420,531	European	Age, sex and the first 10 genetic principal components	UK Biobank (26)	25826379
	Barrett's esophagus	-	361,194	European	Age, sex and the first 10 genetic principal components	UK Biobank (26)	25826379
	Gastroesophageal reflux disease	-	420,531	European	Age, sex and the first 10 genetic principal components	UK Biobank (26)	25826379
Educational attainment	Number of years in education	SD increase in years of education	766,345	European	Age, sex, and the first 10 principal components	Lee et al. (21)	30038396

### 3.2. Effects of educational attainment on EC, BE, and GERD

**Figure 3:**
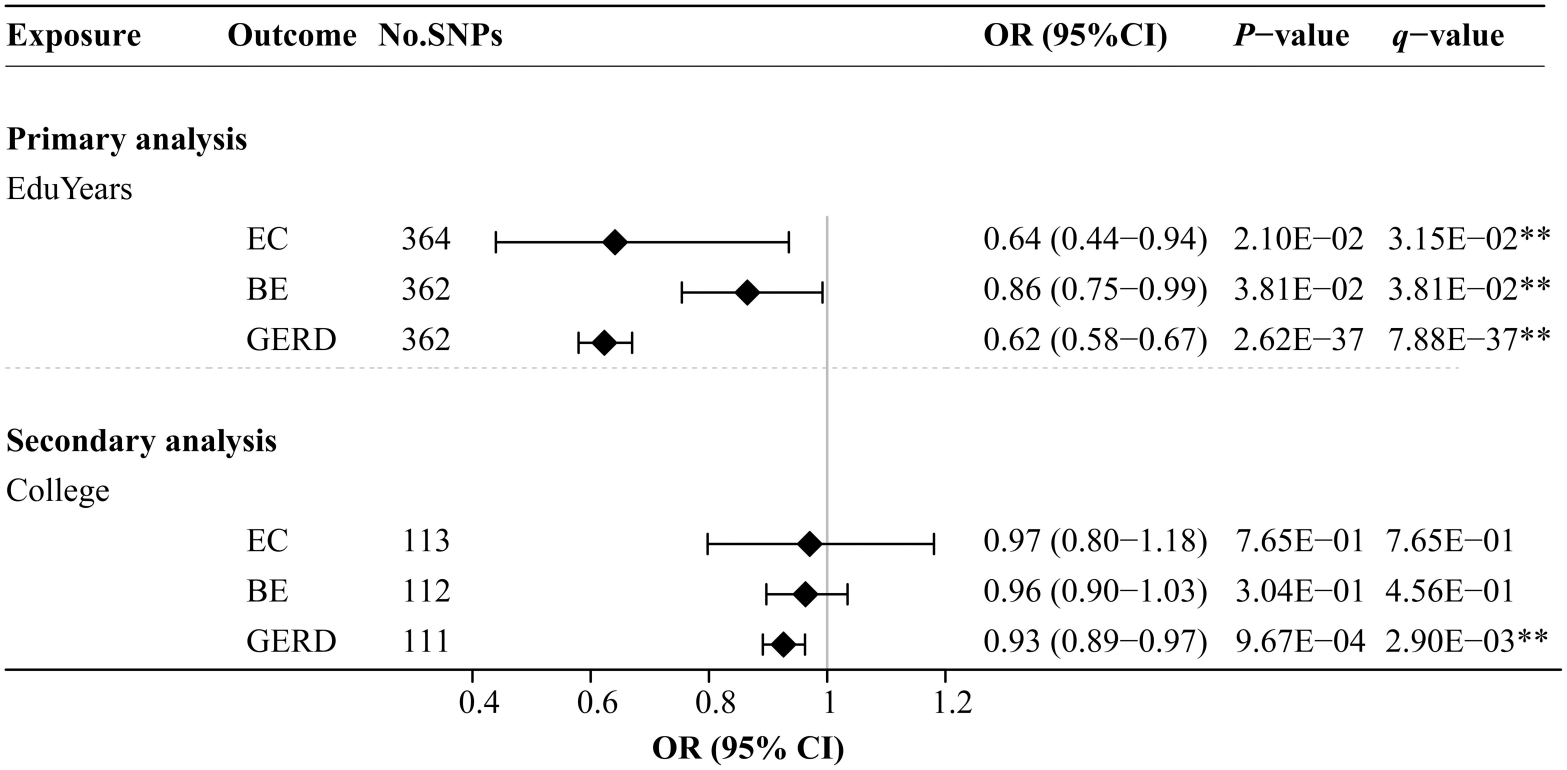
Univariable MR analysis results of the causal effects of education traits on esophageal diseases. Odds ratios were scaled genetically predicted per 3.6 years increase in years of education. Odds ratios were scaled per genetically predicted 1-unit-higher log-odds of liability to college completion (per 2.72-fold increase in the rate of college completion). **Indicates significant evidence (*q*-value < 0.05) after correction for multiple testing estimated by the FDR method.

Figure 3 and Supplementary Table S5 show the effects of genetically predicted two educational traits on EC, BE, and GERD by univariable MR analyses. The MR-Egger intercept test confirmed that there was no significant horizontal pleiotropy (*P* > 0.05). Genetically predicted each SD (3.6 years) increase in EduYears was associated with significantly decreased risks of EC [odds ratio (OR) 0.64; 95% confidence interval (CI) 0.44–0.94], BE (OR 0.86; 95% CI 0.75–0.99), and GERD (OR 0.62; 95% CI 0.58–0.67). Secondary analysis results showed that genetically predicted 1-unit-higher log-odds of liability to college completion were associated with significantly decreased risks of GERD (OR 0.93; 95% CI 0.89–0.97). However, there was no evidence that college completion significantly decreased the risk of EC and BE.

### 3.3. Effects of educational attainment on modifiable risk factors

**Figure 4:**
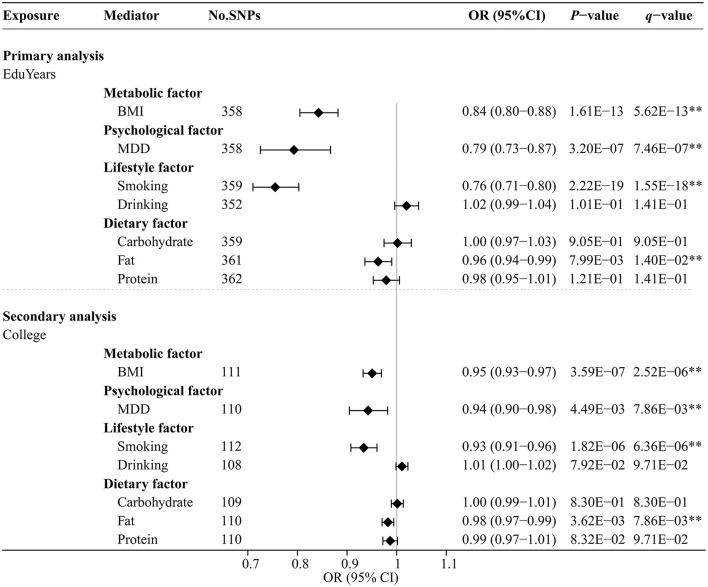
Univariable MR analysis results of the causal effects of education traits on modifiable risk factors. Odds ratios were per 3.6 years or unit change in modifiable risk factor scaled per genetically predicted SD increase in years of education and per genetically predicted 1-unit-higher log-odds of liability to college completion (per 2.72-fold increase in the rate of college completion). **Indicates significant evidence (*q*-value < 0.05) after correction for multiple testing estimated by the FDR method.

Figure 4 and Supplementary Table S6 show the effects of genetically predicted two educational attainment traits and eight modifiable risk factors by univariable MR analyses. The MR-Egger intercept test confirmed that there was no significant horizontal pleiotropy (*P* > 0.05). Genetically predicted EduYears was significantly negatively associated with four risk factors, including BMI (OR 0.84; 95% CI 0.80–0.88), MDD (OR 0.79; 95% CI 0.73–0.87), cigarettes smoked per day (OR 0.76; 95% CI 0.71–0.80), and fat intake (OR 0.96; 95% CI 0.94–0.99). Similarly, secondary analysis results showed that genetically predicted College was significantly negatively associated with four risk factors, including BMI (OR 0.95; 95% CI 0.93–0.97), MDD (OR 0.94; 95% CI 0.90–0.98), cigarettes smoked per day (OR 0.93; 95% CI 0.91–0.96), and fat intake (OR 0.98; 95% CI 0.97–0.99).

### 3.4. Effects of modifiable risk factors on EC, BE, and GERD after adjusting education attainment

**Figure 5:**
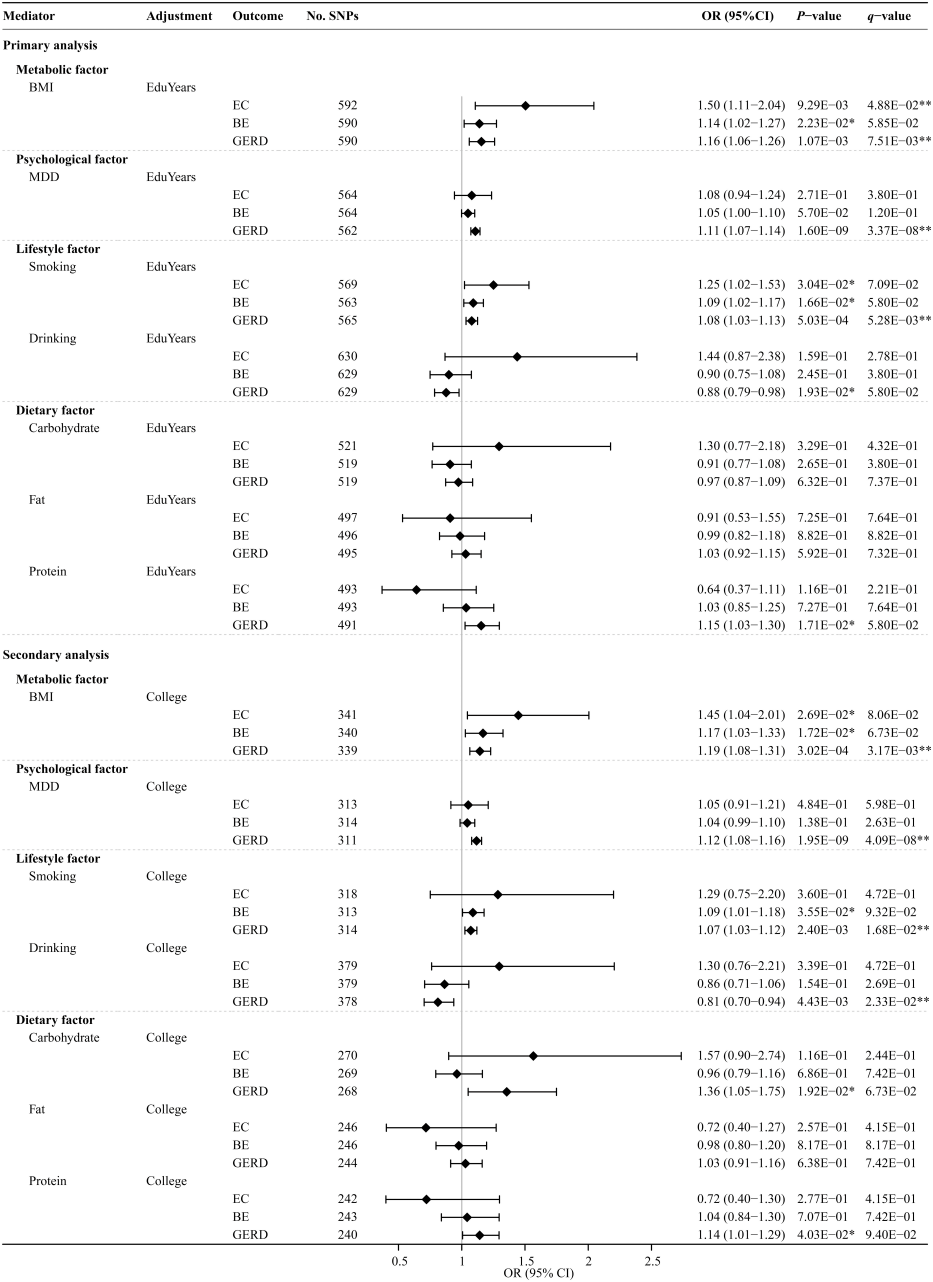
Multivariable MR analysis results of the causal effects of modifiable risk factors on esophageal diseases after adjusting education. Odds ratios were scaled per 3.6 years increase in BMI, smoking, drinking, carbohydrate, fat, and protein. Odds ratios were scaled per genetically predicted 1-unit-higher log-odds of liability to MDD (per 2.72-fold increase in the prevalence). **Indicates significant evidence (*q*-value < 0.05) after correction for multiple testing estimated by the FDR method. *Indicates suggestive evidence (uncorrected *P*-value < 0.05 and *q*-value ≥ 0.05).

The UVMR analysis results of the effects of modifiable risk factors on EC, BE, and GERD are shown in Supplementary Table S7. MR-estimated effects of each modifiable risk factor separately on EC, BE, and GERD after MVMR adjustment for education traits are shown in Figure 5 and Supplementary Table S8. The multivariable MR-Egger intercept test confirmed that there was no significant horizontal pleiotropy (*P* > 0.05). Genetically predicted BMI significantly or suggestively increased the risks of EC, BE, and GERD [adjusting for EduYears: OR, 1.50 (95% CI, 1.11–2.04), 1.14 (95% CI, 1.02–1.27), and 1.16 (95% CI, 1.06–1.26); adjusting for College: OR, 1.45 (95% CI, 1.04–2.01), 1.17 (95% CI, 1.03–1.33), and 1.19 (95% CI, 1.08–1.31)]. Genetically predicted MDD significantly increased the risks of GERD [adjusting for EduYears: OR, 1.11 (95% CI, 1.07–1.14); adjusting for College: OR, 1.12 (95% CI, 1.08–1.16)]. Genetically predicted smoking significantly or suggestively increased the risks of EC, BE, and GERD [adjusting for EduYears: OR, 1.25 (95% CI, 1.02–1.53), 1.09 (95% CI, 1.02–1.17), and 1.08 (95% CI, 1.03–1.13)].

### 3.5. Mediation analysis by modifiable risk factors

**Figure 6:**
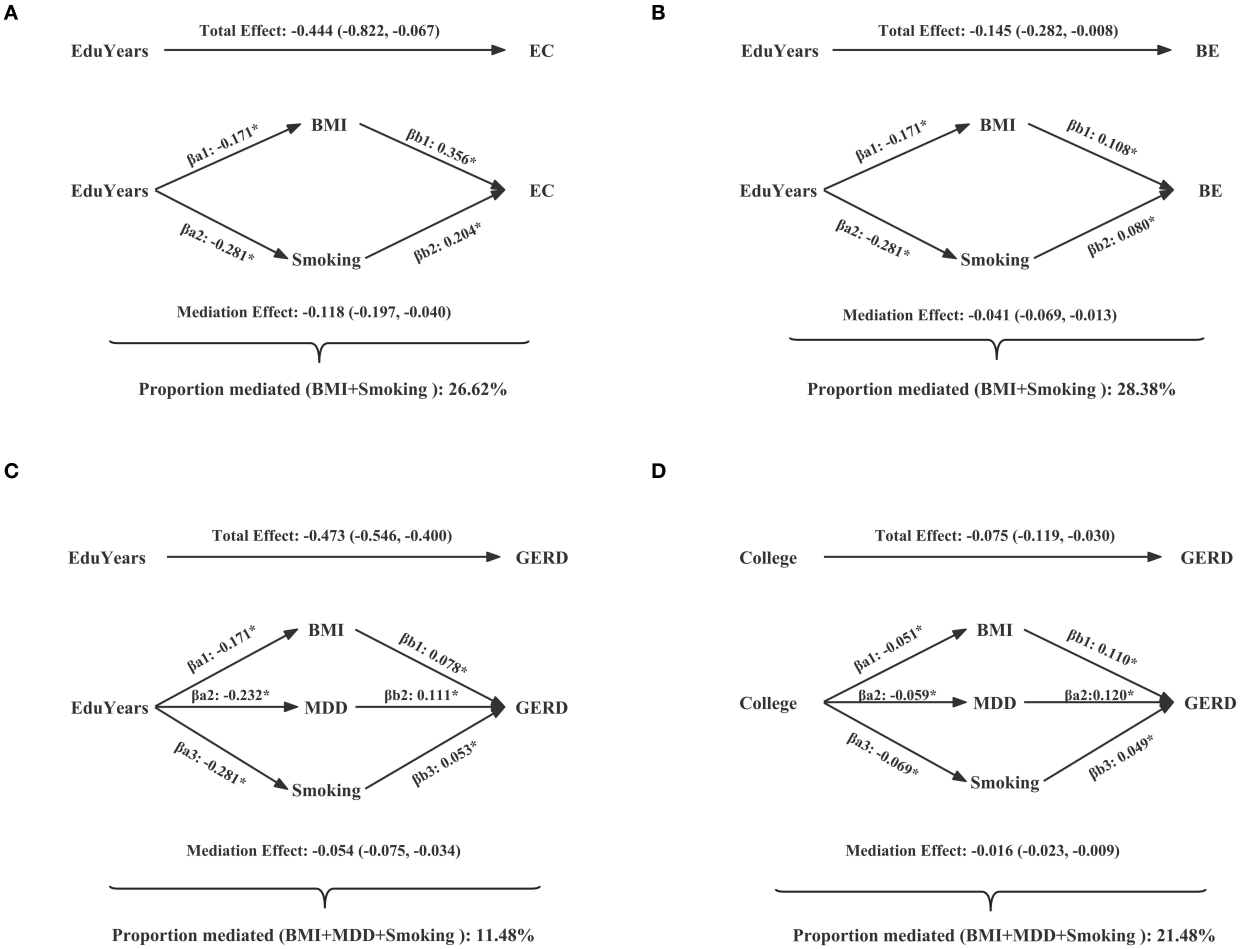
Combined proportions mediated by multiple mediators on EC, BE, and GERD. **(A)** The combined proportions mediated by BMI and smoking for the effect of EduYears on EC. **(B)** The combined proportions mediated by BMI and smoking for the effect of EduYears on BE. **(C)** The combined proportions mediated by BMI, MDD, and smoking for the effect of EduYears on GERD. **(D)** The combined proportions mediated by BMI, MDD, and smoking for the effect of College on GERD.

Table 2 and Supplementary Table S9 show the mediated effect of education on EC, BE, and GERD explained by each mediator separately. The results showed that the effect of EduYears on the risk of EC was mediated by BMI (proportion mediated 15.75%; 95% CI 3.20–28.29%) and smoking (proportion mediated 14.15%; 95% CI 1.01–27.29%). The effect of EduYears on the risk of BE was mediated by BMI (proportion mediated 15.46%; 95% CI 1.61–29.30%) and smoking (proportion mediated 16.85%; 95% CI 2.63–31.08%). The effect of EduYears on the risk of GERD was mediated by BMI (proportion mediated 5.23%; 95% CI 1.80–8.66%), smoking (proportion mediated 4.49%; 95% CI 1.79–7.19%), and MDD (proportion mediated 4.98%; 95% CI 2.48–7.48%). Secondary analysis results showed that the effect of College on the risk of GERD was mediated by BMI (proportion mediated 11.72%; 95% CI 3.92–19.51%), MDD (proportion mediated 6.17%; 95% CI 1.51–10.82%), and smoking (proportion mediated 8.77%; 95% CI 2.15–15.39%). The combined proportions mediated by multiple mediators on EC, BE, and GERD are shown in Figure 6 and Supplementary Table S10. The combined proportions mediated by BMI and smoking for the effect of EduYears on EC and BE were 26.62 and 28.38%. The combined proportions mediated by BMI, MDD, and smoking for the effect of EduYears and College on GERD were 11.48 and 21.48%.

**Table 2:** Estimated proportion mediated for the effect of education on EC, BE, and GERD explained by each mediator separately.

**Exposure**	**Mediator**	**Outcome**	Total effect: βc (95% CI)^*^	**Direct effect: βa (95% CI)^†^**	**Direct effect: βb (95% CI)^‡^**	**Mediation effect**		**Proportion mediated (95%CI)**
						**Effect size (95% CI)**	***P*-value**	
**Primary analysis**								
EduYears	BMI	EC	−0.444 (−0.822, −0.067)	−0.171 (−0.217, −0.126)	0.408 (0.102, 0.715)	−0.070 (−0.126, −0.014)	1.39E-02	15.75 (3.20–28.29)
EduYears	Smoking	EC	−0.444 (−0.822, −0.067)	−0.281 (−0.342, −0.22)	0.224 (0.022, 0.426)	−0.063 (−0.121, −0.004)	3.49E-02	14.15 (1.01–27.29)
EduYears	BMI	BE	−0.145 (−0.282, −0.008)	−0.171 (−0.217, −0.126)	0.131 (0.019, 0.243)	−0.022 (−0.042, −0.002)	2.87E-02	15.46 (1.61–29.30)
EduYears	Smoking	BE	−0.145 (−0.282, −0.008)	−0.281 (−0.342, −0.220)	0.087 (0.016, 0.158)	−0.024 (−0.045, −0.004)	2.02E-02	16.85 (2.63–31.08)
EduYears	BMI	GERD	−0.473 (−0.546, −0.400)	−0.171 (−0.217, −0.126)	0.144 (0.028, 0.165)	−0.025 (−0.041, −0.009)	2.79E-03	5.23 (1.80–8.66)
EduYears	Smoking	GERD	−0.473 (−0.546, −0.400)	−0.281 (−0.342, −0.220)	0.076 (0.033, 0.118)	−0.021 (−0.034, −0.008)	1.11E-03	4.49 (1.79–7.19)
EduYears	MDD	GERD	−0.473 (−0.546, −0.400)	−0.232 (−0.321, −0.143)	0.102 (0.069, 0.134)	−0.024 (−0.035, −0.012)	9.17E-05	4.98 (2.48–7.48)
**Secondary analysis**								
College	BMI	GERD	−0.075 (−0.119, −0.030)	−0.051 (−0.07, −0.031)	0.173 (0.062, 0.206)	−0.009 (−0.015, −0.003)	3.22E-03	11.72 (3.92–19.51)
College	Smoking	GERD	−0.075 (−0.119, −0.030)	−0.069 (−0.097, −0.040)	0.067 (0.025, 0.110)	−0.005 (−0.008, −0.001)	9.43E-03	6.17 (1.51–10.82)
College	MDD	GERD	−0.075 (−0.119, −0.030)	−0.059 (−0.100, −0.018)	0.111 (0.077, 0.144)	−0.007 (−0.012, −0.002)	9.40E-03	8.77 (2.15–15.39)

* Total effect βc: the effect of educational attainment on the risk of EC, BE, and GERD.

† Direct effect βa: the effect of educational attainment on the risk of modifiable risk factors.

‡ Direct effect βb: the effect of modifiable risk factors on the risk of EC, BE, and GERD after adjusting two educational attainment traits.

### 3.6. Sensitivity analysis

The robustness of the main results through a variety of supplementary MR analyses was verified (Supplementary Tables S11–S13). The results showed that the maximum likelihood method and median method (including weighted median and penalized weighted median) showed good consistency with the main analysis results, and the effect size and direction of the MR-Egger method were consistent with the main analysis results, but some may not be statistically significant due to less precision of MR-Egger method. The results of the radial IVW method (Supplementary Tables S14–S16) and re-performed MR analysis under a strict threshold (Supplementary Tables S17–S19) were consistent with the main analysis results, indicating an absence of weak instrument bias. To verify the representativeness of the IVs of EduYears in the main analysis, the MR analysis was repeated using education with the largest GWAS (but with a large overlap with the outcome sample), and the results were consistent with the main analysis (Supplementary Tables S20, S21).

## 4. Discussion

To the best of our knowledge, this is the first comprehensive MR study to examine the causal effects of education on EC, BE, and GERD, and to access the potential mediational effect of modifiable risk factors on the causal association between education and EC, BE, and GERD. Our results suggested that genetically determined higher levels of education were causally associated with decreased risk of EC, BE, and GERD. In addition, we found that genetically predicted higher levels of education were causally associated with lower BMI and lower risk of depression, as well as the lower frequency of smoking. In the aforementioned causality, BMI and smoking mediated 15.75 and 14.15% of the total effect of EduYears on EC, and 15.46 and 16.85% of the total effect of EduYears on BE. The effect of EduYears on the risk of GERD was mediated by BMI, MDD, and smoking, with the proportion mediated at 5.23, 4.98, and 4.49%. For combined mediation, the aforementioned mediators explained 26.62, 28.38, and 11.48% of the effect of EduYears on EC, BE, and GERD.

Although observational studies are inconsistent, most studies suggest that years in education are a protective factor for EC, BE, and GERD. For EC and BE, Thrift et al. (5) and Lagergren et al. (57) found that the risk of EC and BE decreased with the increase of years in education. Notably, since college completion in the secondary analysis is a classification variable, it is different from the content and meaning reflected by the continuous variable (EduYears) in the primary analysis. In addition, the classification variable may also produce different results due to different classification thresholds. This may lead to the fact that our secondary analysis did not find that college completion significantly reduced the risk of EC and BE. Similarly, the EPIC cohort study (*N* = 425,613) showed that college completion does not significantly reduce the risk of EC (58). For GERD, HUNT2 (1995–1997) and HUNT3 (2006–2008) studies showed higher education population had a lower relative risk of GERD (6, 59). The aforementioned results are observational studies, and there have been few MR studies of causal association education with EC, BE, and GERD at the genetic level. Our study suggested for genetically predicted each SD (3.6 years) increase in educational attainment, the relative odds of EC, BE, and GERD were 36, 14, and 38% lower. Although the aforementioned several observational studies indicated the protective effect sizes of higher educational attainment on EC attenuate with adjustment for modifiable risk factors, this suggests a mediation role. However, none of them formally considered a mediation approach to quantify the relative contribution of these factors at the genetic level.

Several MR studies investigating the causal effects of modifiable risk factors on outcomes were roughly consistent with our results. For BMI, Thrift et al. found genetically predicted EAC and BE risk increased by 16 and 12% per 1 kg/m^2^ increase in BMI (60). Our study demonstrates that BMI remains a risk factor after adjusting for education traits. For MDD, the GWAS study of Ong et al. showed that SNP associated with depressive symptoms are not strong predictors of EAC and BE (61). We have not found any MR study of MDD in relation to EC and BE. Wu et al. found GERD risk increased by 1.23-fold with per SD increase in MDD consistent with our result (62). For smoking, Larsson et al. found that genetic predisposition to smoking initiation was associated with statistically significant higher odds of EC (OR 1.83; 95% CI 1.34–2.49) (63). However, Green et al. found that BMI and smoking did not significantly increase the risk of GRED, which may be due to the bias caused by a few IVs of BMI (*n* = 72) and cigarettes per day (*n* = 4) with low explained variation and statistical power (64). For drinking, Zhou et al. found that higher education attainment was significantly associated with alcohol intake in the UK (65). However, considering the overlap of the study samples, which may increase the risk of type I errors, the author's interpretation of this positive result needs to be cautious. Notably, Rosoff et al. used exposure and outcome without sample overlap and found no significant association between education and weekly alcohol consumption, which is consistent with our results (66).

Several observational studies supported mediating roles of modifiable risk factors between education and the risk of cancer. In the Canadian Community Health Survey, Nejatinamini et al. attributed almost 45% of the association between low SEP (combining education and household income) and overall cancer morbidity and mortality to modifiable risk factors, among them, obesity, smoking, excess alcohol intake, and low fruit-and-vegetable intake accounted for 11.6, 15.5, 7.7, and 7.7% (10). This suggests that public health interventions to reduce excess adiposity, mental health burden, and smoking would have widespread benefits on cancer. In our analysis, the majority proportion of the effect of education remains unaccounted for. The proportion of the total effect of EduYears on EC and BE mediated by BMI or smoking was about 15%. BMI, MDD, or smoking only mediated about 5% of the total effect of EduYears on GERD. The possible explanation is that GERD is a more common disease than cancer and is, thus, susceptible to more extensive factors in a short period of time. In addition, the pathophysiology of GERD is complex, and several factors have been implicated, including diminished gastric volume, increased intragastric pressure, and decreased lower esophageal sphincter pressure.

Educational attainment is a proxy for overall SEP because it is associated with both income and occupation later in life (8). Low education and low SEP confer vulnerability to esophageal diseases and probably in part by diminishing access to economic, cultural, and social resources, whose reduction are relevant to risk factors for smoking, alcohol intake, dietary intake, body weight, and mental health disorders (67, 68). Our study may provide some clinical and public health implications. First, this is a comprehensive MR study to support the causal association between high education and low risk of esophageal cancer and its precancerous lesions, avoiding reverse causality and residual confounding factors in observational studies. This discovery suggests that it is necessary to strengthen investment in education intervention for the prevention of esophageal diseases. Second, given the difficulty and long timelines required to implement upstream interventions for educational inequality, for those who cannot change their education level at present, midstream interventions for modifiable risk factors are more practicable to reduce esophageal diseases. Specifically, on the one hand, for people with low education levels, publicity and health science popularization can increase their attention to risk factors such as high BMI, smoking, and psychological, allowing them to control or reduce these risk factors from an individual perspective. On the other hand, in rural or disadvantaged communities, midstream interventions such as locating recreation and sports facilities, setting up smoke-free spaces, and establishing psychological counseling centers could mitigate the impact of these mediating risk factors on people with low education. This has important implications for reducing inequality in education on esophageal cancer risk in the short term.

There are three main strengths in the present study. First, the use of SNPs as a genetic tool reduces bias due to confounding and reverse causality, thereby providing precise causal estimates of the effect of education on outcomes. For example, observational studies are susceptible to socioeconomic and other environmental factors when exploring the association between education and esophageal diseases. Mendelian randomization with genetic variants as instrumental variables is generally not susceptible to confounding by behavioral, socioeconomic, and physiological factors (69). Second, the two-step MR analysis was used to explore the mediated proportions of modifiable risk factors in the foregoing associations, which further provides robustness to non-differential measurement error in the mediator (18). Finally, the relatively consistent results of the two phenotypes of measuring the level of education and sensitivity analyses demonstrate the reliability of our findings.

However, there are several limitations to this study. First, although educational attainment is often used as a proxy for SEP, they are not interchangeable. SEP encompasses more factors than education. The results of other SEP factors may be different from our results. However, educational attainment is largely determined early in life and comparatively easy to measure, while other SEP factors including occupation and income may be influenced by disability at work or other factors (8). Second, due to the limitations of the database availability, on the one hand, based on the education of summary level data, we cannot evaluate the nonlinear relationship between education and outcomes. On the other hand, we can only collect the genetic data of patients with EC, but there is a lack of information about individual histological subtypes. However, we can infer from the tumor site that EAC accounts for about 80% of patients with EC in the UK database. Therefore, our conclusion is more suitable to explain the effect factors of patients with EAC. Third, an inherent limitation of MR analysis is that there may be potential polymorphic effects, but we have removed SNPs associated with pleiotropic outliers detected by the MR-PRESSO method, and the MR-Egger intercept test was used to estimate pleiotropic to prevent bias caused by pleiotropic as much as possible. Fourth, the screening of some IVs in our study relaxed the threshold standard, which may produce weak IV bias; however, this is also to increase the representativeness of IVs and statistical power, and we have verified our results through F-statistics, radial IVW MR analysis method, and sensitivity analysis under strict threshold standard (*P* < 5e-8). Fifth, the statistical power for the outcomes may be limited in the present study. On the one hand, to avoid sample overlap bias, EduYears was derived from the GWAS without UK Biobank individuals. However, the representativeness of the IVs of EduYears had been verified in the sensitivity analysis. On the other hand, due to the limitations of the UK Biobank data, the number of esophageal cancer cases is relatively small; thus, further studies of EC with larger sample GWASs will help to confirm the precision of our results. Finally, the current MR method for mediation analysis assumes a linear association between the exposure, mediator, and outcome (70–72), which is supported by previous studies (4–6, 13, 59, 73–76). In addition, it assumes no interaction between the exposure and mediator, which requires further investigation through observational studies to explore potential interactions between education and modifiable risk factors or the development of MR methods that can account for these interactions.

## 5. Conclusion

Our MR study supports a protective effect of higher educational attainment on the risk of EC and its precancerous lesion and disease including BE and GERD. Moreover, our study provides genetic evidence that the causal effect of educational attainment on EC, BE, and GERD is partly mediated by BMI, smoking, and MDD. These findings demonstrate that improving metabolic, lifestyle, and psychological factors in low-education populations will have important public health significance for the prevention of EC. However, the majority proportion of education's effect on esophageal cancer and its precancerous lesion and disease remains unexplained. Further research into potentially modifiable mediators is necessary.

## Data availability statement

The original contributions presented in the study are included in the article/Supplementary material, further inquiries can be directed to the corresponding author.

## Author contributions

XZ and ML conceived and designed the project and interpreted the results. XZ analyzed and cleaned the data and wrote the manuscript. XYa, TZ, XYi, JM, and ML critically reviewed and revised the manuscript. All authors have approved the final manuscript.
