# Supplementary material for: Association of educational attainment with esophageal cancer, Barrett's esophagus, and gastroesophageal reflux disease, and the mediating role of modifiable risk factors: A Mendelian randomization study
Source: Front Public Health. 2023 Mar 28;11:1022367. doi: 10.3389/fpubh.2023.1022367 (PMC10086429; doi:10.3389/fpubh.2023.1022367)
Supplement: Supplementary file 1 [file Data_Sheet_1.docx]

**Supplementary material**

**Index**

[eMethods 1 Supplemental methods for GWAS data sources of exposure, mediator, and outcome 1](#_Toc105491748)

[eMethods 2 Supplemental methods for Mediation analysis 1](#_Toc105491749)

[Table S1 Description of participating cohorts for EduYears (Okbay et al). 1](#_Toc129556960)

[Table S2 Preliminary screening of candidate SNPs of instrumental variables. 2](#_Toc129556961)

[Table S3 Screening process of final SNPs of instrumental variables for each MR analysis. 3](#_Toc129556962)

[Table S4 Details of removed SNPs. 5](#_Toc129556963)

[Table S5 The univariable MR analysis results of the causal effects of education traits on EC, BE, and GERD. 8](#_Toc129556964)

[Table S6 The univariable MR analysis results of the causal effects of education traits on modifiable risk factors. 8](#_Toc129556965)

[Table S7 The univariable MR analysis results of the causal effects of modifiable risk factors on EC, BE, and GERD. 10](#_Toc129556966)

[Table S8 The multivariable MR analysis results of the single causal effects of individual modifiable risk factors on EC, BE, and GERD after adjusting for education traits. 11](#_Toc129556967)

[Table S9 The results of the mediation effect test that without statistically significant. 14](#_Toc129556968)

[Table S10 The multivariable MR analysis results of the combined causal effects of several modifiable risk factors on EC, BE, and GERD after adjusting for education traits. 15](#_Toc129556969)

[Table S11 The complementary MR analysis results of the causal effects of education traits on EC, BE, and GERD. 17](#_Toc129556970)

[Table S12 The complementary MR analysis results of the causal effects of education traits on modifiable risk factors. 17](#_Toc129556971)

[Table S13 The complementary MR analysis results of the causal effects of modifiable risk factors on EC, BE, and GERD. 18](#_Toc129556972)

[Table S14 Verification results of potentially weak instrumental bias in causal effects of education traits on EC, BE, and GERD. 21](#_Toc129556973)

[Table S15 Verification results of potentially weak instrumental bias in causal effects of education traits on modifiable risk factors. 21](#_Toc129556974)

[Table S16 Verification results of potentially weak instrumental bias in causal effects of modifiable risk factors on EC, BE, and GERD. 21](#_Toc129556975)

[Table S17 MR analysis results of the effect of instrumental variables of education traits on EC, BE, and GERD screened by strict threshold criteria (P < 5e-8). 22](#_Toc129556976)

[Table S18 MR analysis results of the effect of instrumental variables of education traits on modifiable risk factors screened by strict threshold criteria (P < 5e-8). 22](#_Toc129556977)

[Table S19 MR analysis results of the effect of instrumental variables of modifiable risk factors on EC, BE, and GERD screened by strict threshold criteria (P < 5e-8). 23](#_Toc129556978)

[Table S20 MR analysis results of the effect of instrumental variables of education traits on EC, BE, and GERD (using GWAS including UK Biobank individuals, Lee et al.) on EC, BE, and GERD. 25](#_Toc129556979)

[Table S21 MR analysis results of the effect of instrumental variables of education traits on modifiable risk factors (using GWAS including UK Biobank individuals, Lee et al.) on modifiable risk factors. 25](#_Toc129556980)

## eMethods 1 Supplemental methods for GWAS data sources of exposure, mediator, and outcome

### GWAS data for exposure

Genetic association results for educational attainment were identified from GWAS meta-analysis of SSGAC consortium. To create a harmonized measure of educational attainment, Rietveld et al. coded study-specific measures using the International Standard Classification of Education (ISCED, 1997) scale. We analyzed a quantitative variable defined as an individual’s years of schooling (EduYears) and a binary variable for college completion (College).

EduYears was measured by the number of years of full-time education. Respondents from 65 cohorts reported the levels of educational qualifications attained. The highest educational qualification was converted into number of years in education as variable EduYears using the ISCED category ^1^. Data from 65 studies were harmonized against the ISCED (1997) classification system (see supplementary table 1 of the original GWAS study).

College is the binary variable that distinguishes between those with a tertiary degree and those without. This binary variable was imputed taking the value 1 if the individual had completed a college degree (ISCED level 5 or above of the ISCED classification), and 0 if the individual had not completed a college degree (ISCED level 4 or below) ^2^.

## Table S1 Description of participating cohorts for EduYears (Okbay et al).

| Study | Full name | Sampling | Country | Sample size | Birth year (Mean/Range) | Fraction female |
| --- | --- | --- | --- | --- | --- | --- |
| ACPRC | Manchester Studies of Cognitive Ageing | Population-based | England | 1713 | 1923 | 0.71 |
|  |  |  |  |  | (1903-1948) |  |
| AGES | Age, Gene/ Environment Susceptibility–Reykjavik Study | Population-based | Iceland | 3212 | 1927 | 0.58 |
|  |  |  |  |  | (1908-1936) |  |
| ALSPAC | Avon Longitudinal Study of Parents and Children | Population-based birth cohort | England | 2877 | 1959 | 1 |
|  |  |  |  |  | (1948-1963) |  |
| ASPS | Austrian Stroke Prevention Study | Population-based | Austria | 777 | 1932 | 0.57 |
|  |  |  |  |  | (1909-1949) |  |
| BASE-II | Berlin Aging Study II | Population-based | Germany | 1619 | 1948 | 0.52 |
|  |  |  |  |  | (1925-1983) |  |
| CoLaus | Cohorte Lausannoise | Population-based | Switzerland | 3269 | 1950 | 0.53 |
|  |  |  |  |  | (1928-1970) |  |
| COPSAC2000 | Copenhagen Studies on Asthma in Childhood 2000 | Case-control birth cohort | Germany | 318 | 1966 | 0.47 |
|  |  |  |  |  | (1964-1969) |  |
| CROATIA-Korčula | Croatia Korčula | Population-based (Isolate) | Croatia | 842 | 1950 | 0.64 |
|  |  |  |  |  | (1909-1977) |  |
| deCODE | deCODE genetics | Population-based | Iceland | 46758 | 1945 | 0.57 |
|  |  |  |  |  | (1894-1983) |  |
| DHS | Dortmund Health Study | Population-based | Germany | 953 | 1949 | 0.53 |
|  |  |  |  |  | (1929-1974) |  |
| DIL | Wellcome Trust Diabetes and Inflammation Laboratory | Population-based | England | 2578 | 1958 | 0.52 |
|  |  |  |  |  | (1958-1958) |  |
| EGCUT1 | Estonian Genome Center, University of Tartu | Population-based | Estonia | 5597 | 1950 | 0.55 |
|  |  |  |  |  | (1905-1980) |  |
| EGCUT2 | Same as above | Population-based | Estonia | 1328 | 1957 | 0.53 |
|  |  |  |  |  | (1911-1979) |  |
| EGCUT3 | Same as above | Population-based | Estonia | 2047 | 1966 | 0.73 |
|  |  |  |  |  | (1930-1982) |  |
| ERF | Erasmus Rucphen Family Study | Family-based | Netherlands | 2433 | 1952 | 0.55 |
|  |  |  |  |  | (1914-1974) |  |
| FamHS | Family Heart Study | Family-based | USA | 3483 | 1941 | 0.53 |
|  |  |  |  |  | (1900-1965) |  |
| FINRISK | The National FINRISK Study | Case-control (Cardiovascular health) | Finland | 1685 | 1946 | 0.46 |
|  |  |  |  |  | (1923-1977) |  |
| FTC | Finnish Twin Cohort | Family-based | Finland | 2418 | 1945 | 0.56 |
|  |  |  |  |  | (1910-1972) |  |
| GOYA | Genetics of Overweight Young Adults | Case-control (Obesity) | Denmark | 1459 | 1947 | 0 |
|  |  |  |  |  | (1944-1954) |  |
| GRAPHIC | Genetic Regulation of Arterial Pressure in Humans | Population-based | England | 727 | 1951 | 0.53 |
|  |  |  |  |  | (1942-1965) |  |
| GS | Generation Scotland | Population-based | Scotland | 8776 | 1955 | 0.59 |
|  |  |  |  |  | (1909-1981) |  |
| H2000 Cases | Health 2000 | Case-control (Metabolic syndrome) | Finland | 797 | 1949 | 0.5 |
|  |  |  |  |  | (1924-1970) |  |
| H2000 Controls | Same as above | Case-control (Metabolic syndrome) | Finland | 819 | 1949 | 0.52 |
|  |  |  |  |  | (1924-1969) |  |
| HBCS | Helsinki Birth Cohort Study | Population-based birth cohort | Finland | 1617 | 1941 (1934-1944) | 0.57 |
| HCS | Hunter Community Study | Population-based | Australia | 1946 | 1940 | 0.49 |
|  |  |  |  |  | (1920-1951) |  |
| HNRS (CorexB) | Heinz Nixdorf Recall Study | Population-based | Germany | 1401 | 1942 | 0.5 |
|  |  |  |  |  | (1926-1955) |  |
| HNRS (Oexpr) | Same as above | Same as above | Germany | 1347 | 1942 | 0.5 |
|  |  |  |  |  | (1926-1955) |  |
| HNRS (Omni1) | Same as above | Same as above | Germany | 778 | 1942 | 0.52 |
|  |  |  |  |  | (1927-1955) |  |
| HRS | Health and Retirement Study | Population-based | USA | 9963 | 1940 | 0.42 |
|  |  |  |  |  | (1900-1979) |  |
| Hypergenes | Hypergenes | Case-control | Italy/ UK/ Belgium | 815 | 1945 | 0.46 |
|  |  |  |  |  | (1914-1971) |  |
| INGI-CARL | Italian Network of Genetic Isolates - Carlantino | Population-based (Isolate) | Italy | 947 | 1946 | 0.58 |
|  |  |  |  |  | (1910-1975) |  |
| INGI-FVG | Italian Network of Genetic Isolates - Friuli Venezia Giulia | Population-based (Isolate) | Italy | 943 | 1951 | 0.6 |
|  |  |  |  |  | (1917-1978) |  |
| KORA S3 | Kooperative Gesundheitsforschung in der Region Augsburg | Population-based | Germany | 2655 | 1945 | 0.51 |
|  |  |  |  |  | (1920-1964) |  |
| KORA S4 | Same as above | Population-based | Germany | 2721 | 1949 | 0.51 |
|  |  |  |  |  | (1926-1970) |  |
| LBC1921 | Lothian Birth Cohort 1921 | Population-based birth cohort | Scotland | 515 | 1921 | 0.58 |
|  |  |  |  |  | (1921-1921) |  |
| LBC1936 | Lothian Birth Cohort 1936 | Population-based birth cohort | Scotland | 1003 | 1936 | 0.49 |
|  |  |  |  |  | (1936-1936) |  |
| LifeLines | The LifeLines Cohort Study | Population-based | Netherlands | 12539 | 1960 | 0.58 |
|  |  |  |  |  | (1921-1980) |  |
| MCTFR | Minnesota Center for Twin and Family Research | Family-based, but only founders used. | USA | 3819 | 1953 | 0.54 |
|  |  |  |  |  | (1926-1974) |  |
| MGS | Molecular Genetics of Schizophrenia | Population-based | USA | 2313 | 1951 | 0.5 |
|  |  |  |  |  | (1914-1976) |  |
| MoBa | Mother and Child Cohort of NIPH | Population-based (Nested case-control) | Norway | 622 | 1971 | 1 |
|  |  |  |  |  | (1966-1976) |  |
| NBS | Nijmegen Biomedical Study | Population-based | Netherlands | 1808 | 1941 | 0.5 |
|  |  |  |  |  | (1923-1972) |  |
| NESDA | Netherlands Study of Depression and Anxiety | Case-control (Mental health) | Netherlands | 1820 | 1958 | 0.64 |
|  |  |  |  |  | (1939-1977) |  |
| NFBC66 | Northern Finland Birth Cohort 1966 | Population-based | Finland | 5297 | 1966 | 0.52 |
|  |  |  |  |  | (1966-1966) |  |
| NTR | Netherlands Twin Register | Family-based | Netherlands | 5246 | 1958 | 0.64 |
|  |  |  |  |  | (1917-1989) |  |
| OGP | Ogliastra Genetic Park | Population-based | Italy | 370 | 1950 | 0 |
|  |  |  |  |  | (1916-1976) |  |
| OGP-Talana | Ogliastra Genetic Park-Talana | Population-based (Isolate) | Italy | 544 | 1949 | 0.59 |
|  |  |  |  |  | (1910-1977) |  |
| ORCADES | Orkney Complex Disease Study | Population-based (Isolate) | Scotland | 1828 | 1952 | 0.6 |
|  |  |  |  |  | (1914-1979) |  |
| PREVEND | Prevention of Renal and Vascular End-stage Disease | Population-based | Netherlands | 3578 | 1948 | 0.48 |
|  |  |  |  |  | (1923-1968) |  |
| QIMR | Queensland Institute of Medical Research | Family-based | Australia | 8006 | 1956 | 0.59 |
|  |  |  |  |  | (1900-1984) |  |
| RS-I | Rotterdam Study Baseline | Population-based | Netherlands | 6108 | 1922 | 0.6 |
|  |  |  |  |  | (1893-1938) |  |
| RS-II | Rotterdam Study Extension of Baseline | Same as above | Netherlands | 1667 | 1935 | 0.52 |
|  |  |  |  |  | (1906-1944) |  |
| RS-III | Rotterdam Study Young | Same as above | Netherlands | 3040 | 1950 | 0.56 |
|  |  |  |  |  | (1910-1960) |  |
| Rush-MAP | Rush University Medical Center - Memory and Aging Project | Community-based | USA | 887 | 1921 | 0.72 |
|  |  |  |  |  | (1901-1948) |  |
| Rush-ROS | Rush University Medical Center - Religious Orders Study | Community-based | USA | 808 | 1921 | 0.66 |
|  |  |  |  |  | (1896-1946) |  |
| SardiNIA | SardiNIA Study of Aging | Family-based | Italy | 5616 | 1955 | 0.58 |
|  |  |  |  |  | (1901-1983) |  |
| SHIP | Study of Health in Pomerania | Population-based | Germany | 3556 | 1945 | 0.5 |
|  |  |  |  |  | (1918-1971) |  |
| SHIP-TREND | Study of Health in Pomerania | Population-based | Germany | 901 | 1956 | 0.57 |
|  |  |  |  |  | (1928-1980) |  |
| STR – Salty | Swedish Twin Registry | Family-based | Sweden | 4832 | 1951 | 0.52 |
|  |  |  |  |  | (1943-1958) |  |
| STR – Twingene | Swedish Twin Registry | Family-based | Sweden | 9553 | 1941 | 0.53 |
|  |  |  |  |  | (1916-1958) |  |
| THISEAS | The Hellenic Study of Interactions between SNPs & Eating in Atherosclerosis Susceptibility | Case-control | Greece | 829 | 1950 | 0.33 |
|  |  |  |  |  | (1909-1979) |  |
| TwinsUK | St Thomas’ UK Adult Twin Registry | Population-based | England | 4012 | 1949 | 1 |
|  |  |  |  |  | (1919-1978) |  |
| WTCCC58C | 1958 British Birth Cohort | Population-based | England | 2804 | 1958 | 0.48 |
|  |  |  |  |  | (1958-1958) |  |
| YFS | The Cardiovascular Risk in Young Finns Study | Population-based | Finland | 2029 | 1969 | 0.55 |
|  |  |  |  |  | (1962-1977) |  |
| 23andMe | 23andMe, inc | Genomics company | Primarily US | 76155 | 1961 | 0.52 |
|  |  |  |  |  | (1901-1985) |  |

### GWAS data for mediator

Genetic association results for BMI were extracted from GWAS meta-analysis (including 82 GWAS and 43 Metabochip studies) published by GIANT Consortium, totaling 322,154 individuals of European ancestry. BMI was calculated as dividing weight by height squared (kg/m2) ^3^.

Genetic association estimates for MDD were derived from GWAS meta-analysis published by PGC consortium based on 173,005 European ancestry (59,851 MDD cases and 113,154 controls) from PGC29 cohort and the five additional cohorts. According to the international recognized Standard (DSM-IV, ICD-9, or ICD-10), MDD patients could be diagnosed through structured interview, clinician management checklist, or review of medical records. The inclusion and exclusion criteria for cases and controls were to cases with exclude lifetime bipolar disorder and to exclude controls with modifiable risk factors ^4^.

Genetic association results for smoking quantity and alcohol consumption were derived from GWAS meta-analysis conducted by GSCAN consortium ^5^. Cigarettes Per Day, a variable of smoking intensity, defined as the average number of cigarettes smoked per day by current or former smokers. Participants (N = 249,752) in GSCAN consortium were asked: “How many cigarettes do/did you smoke per day?” with responses provide any integer. Drinks Per Week defined as the average number of all types of alcohol consumed per week, which reflected common alcohol drinking behavior, not excessive or harmful drinking behavior (mean = 10.8, SD = 1.90). Participants (N = 335,394) in GSCAN consortium were asked: “How many alcoholic beverages did you have in the past week?”. If the respondent provided a range, the midpoint of that range was used. GWAS data for these two phenotypes was left anchored to 1 and log transformed - the phenotype was standardized so that 1 unit increase corresponds to a 1 SD increase.

For three dietary compositions, we utilized the GWAS meta-analysis of European-ancestry individuals (fat/protein/carbohydrate: N = 268,922) released by the SSGAC consortium ^6^. Respondents were surveyed dietary intake with food-frequency questionnaire. Food frequency questionnaire was used to investigate the dietary intake of the respondents and macronutrients intakes were calculated according to the rules.

### GWAS data for outcome

Summary-level genetic data for outcome were obtained from the UK Biobank cohort. Participants were recruited from across the UK and attended one of 22 centers in England, Scotland, and Wales, to provide detailed sociodemographic, health and anthropometric data as well as providing blood and urine samples for subsequent analyses. Participants consented to having their health followed and many have subsequently participated in further monitoring or completed additional questionnaires. The cohort is described in detail elsewhere. Information including questionnaire and nurse interview information, anthropometric and physiological measurements, and genomic data were collected, as described previously. GWAS data for outcome were extracted from a second-round analysis of the UK Biobank data from the Neale Lab ^7^.

## eMethods 2 Supplemental methods for Mediation analysis

Estimate the combined mediation effect through the following steps. First, we used MVMR of multiple parallel mediation analysis to adjust the genetic effect of several mediators simultaneously to obtain each mediation effect of mediators on outcomes under this method. Second, the combined mediation effect is calculated by accumulating each mediation effect under MVMR of multiple parallel mediation analysis, and its standard error calculation is based on the theory of error propagation. Finally, we divided the combined mediated effect by the total effect to estimate the total proportion mediated.

## Table S2 Preliminary screening of candidate SNPs of instrumental variables.

| **Traits** | **Phenotype** | **Strict standard (significant *P*<5e-8)*** | | **Relaxed standard (suggestive *P*<5e-5)*** | |
| --- | --- | --- | --- | --- | --- |
|  |  | **Number of candidate SNPs** | **R2, %** | **Number of candidate SNPs** | **R2, %** |
| **Educational attainment** | EduYears | 73 | 0.95 | 374 | 3.12 |
|  | College | 3 | - | 117 | - |
| **Metabolic factor** | BMI | 69 | 1.47 | 232 | 2.70 |
| **Psychological factor** | MDD | 5 | - | 207 | - |
| **Lifestyle factor** | Smoking | 23 | 0.91 | 210 | 2.40 |
|  | Drinking | 37 | 0.52 | 274 | 1.15 |
| **Dietary factor** | Carbohydrate | 12 | 0.19 | 162 | 1.14 |
|  | Fat | 5 | 0.15 | 138 | 1.13 |
|  | Protein | 7 | 0.15 | 133 | 1.20 |

Abbreviations: EduYears, number of years in education; College, college completion; BMI, body mass index; MDD, major depressive disorder; Smoking, cigarettes smoke per day; Drinking, drinks Per Week; Carbohydrate, Carbohydrate intake; Fat, Fat intake; Protein, Protein intake.

*: Independent SNPs under the two standard were obtained by clustering with linkage disequilibrium (LD) threshold r2 < 0.001 and distance of 10,000kb.

## Table S3 Screening process of final SNPs of instrumental variables for each MR analysis.

| Exposure | Outcome | Number of candidate SNPs, N | Remove SNPs missing in the outcome, N† | Remove ambiguous  palindromic SNPs, N† | Remove horizontal pleiotropic outlier SNPs, N† | Number of final SNPs, N |
| --- | --- | --- | --- | --- | --- | --- |
| EduYears | EC | 374 | 0 | 10 | 0 | 364 |
|  | BE | 374 | 0 | 12 | 0 | 362 |
|  | GERD | 374 | 0 | 12 | 0 | 362 |
| College | EC | 117 | 0 | 4 | 0 | 113 |
|  | BE | 117 | 0 | 5 | 0 | 112 |
|  | GERD | 117 | 0 | 5 | 1 | 111 |
| EduYears | BMI | 374 | 2 | 10 | 4 | 358 |
|  | MDD | 374 | 0 | 11 | 5 | 358 |
|  | Smoking | 374 | 0 | 11 | 4 | 359 |
|  | Drinking | 374 | 2 | 11 | 9 | 352 |
|  | Carbohydrate | 374 | 2 | 10 | 3 | 359 |
|  | Fat | 374 | 2 | 10 | 1 | 361 |
|  | Protein | 374 | 2 | 10 | 1 | 361 |
| College | BMI | 117 | 0 | 3 | 3 | 111 |
|  | MDD | 117 | 0 | 5 | 2 | 110 |
|  | Smoking | 117 | 0 | 5 | 0 | 112 |
|  | Drinking | 117 | 0 | 5 | 4 | 108 |
|  | Carbohydrate | 117 | 1 | 5 | 2 | 109 |
|  | Fat | 117 | 1 | 5 | 1 | 110 |
|  | Protein | 117 | 1 | 5 | 1 | 110 |
| BMI | EC | 232 | 0 | 4 | 0 | 228 |
|  | BE | 232 | 0 | 4 | 0 | 228 |
|  | GERD | 232 | 0 | 4 | 0 | 228 |
| MDD | EC | 207 | 1 | 3 | 3 | 200 |
|  | BE | 207 | 2 | 3 | 0 | 202 |
|  | GERD | 207 | 1 | 3 | 3 | 200 |
| Smoking | EC | 210 | 0 | 5 | 0 | 205 |
|  | BE | 210 | 4 | 5 | 0 | 201 |
|  | GERD | 210 | 1 | 5 | 1 | 203 |
| Drinking | EC | 274 | 0 | 8 | 0 | 266 |
|  | BE | 274 | 1 | 6 | 0 | 267 |
|  | GERD | 274 | 1 | 6 | 0 | 267 |
| Carbohydrate | EC | 162 | 0 | 5 | 0 | 157 |
|  | BE | 162 | 0 | 5 | 0 | 157 |
|  | GERD | 162 | 0 | 5 | 2 | 155 |
| Fat | EC | 138 | 3 | 2 | 0 | 133 |
|  | BE | 138 | 2 | 2 | 0 | 134 |
|  | GERD | 138 | 3 | 2 | 0 | 133 |
| Protein | EC | 133 | 3 | 1 | 0 | 129 |
|  | BE | 133 | 1 | 1 | 0 | 131 |
|  | GERD | 133 | 3 | 1 | 0 | 129 |

†: Detailed SNPs are shown in Supplementary Table 3

## Table S4 Details of removed SNPs.

| Exposure | Outcome | Remove SNPs missing in the outcome | Remove ambiguous palindromic SNPs | Remove horizontal pleiotropic outliers SNPs |
| --- | --- | --- | --- | --- |
| EduYears | EC | NA | rs3768480; rs2477432; rs12134151; rs8036481; rs2135996; rs11130222; rs1121213; rs36136985; rs12534506; rs2980813 | NA |
|  | BE | NA | rs11130222; rs1118399; rs1121213; rs12134151; rs12534506; rs2135996; rs2477432; rs2980813; rs36136985; rs3768480; rs3890065; rs8036481 | NA |
|  | GERD | NA | rs3768480; rs2477432; rs12134151; rs3890065; rs1118399; rs8036481; rs2135996; rs11130222; rs1121213; rs36136985; rs12534506; rs2980813 | NA |
| College | EC | NA | rs238257; rs2280405; rs791903; rs10511431 | NA |
|  | BE | NA | rs10511431; rs16903920; rs2280405; rs238257; rs791903 | NA |
|  | GERD | NA | rs238257; rs2280405; rs16903920; rs791903; rs10511431 | rs17314804 |
| EduYears | BMI | rs139445863; rs7786263 | rs11130222; rs1118399; rs1121213; rs2135996; rs2477432; rs2980813; rs36136985; rs3768480; rs3890065; rs8036481 | rs4807177; rs2031496; rs6882046; rs116475118 |
|  | MDD | NA | rs11130222; rs1118399; rs1121213; rs12134151; rs12534506; rs2135996; rs2477432; rs2980813; rs36136985; rs3768480; rs8036481 | rs1158542; rs12552; rs2431108; rs62100767; rs942862 |
|  | Smoking | NA | rs11130222; rs1121213; rs12134151; rs12534506; rs2135996; rs2477432; rs2980813; rs36136985; rs3768480; rs496590; rs8036481 | rs293570; rs4860729; rs748439; rs57877670 |
|  | Drinking | rs4741351; rs621893 | rs11130222; rs1121213; rs12134151; rs12534506; rs2135996; rs2477432; rs2980813; rs36136985; rs3768480; rs496590; rs8036481 | rs1106761; rs17110109; rs17824247; rs28792186; rs34305371; rs538628; rs62263923; rs8049439; rs1405452 |
|  | Carbohydrate | rs4741351; rs621893 | rs11130222; rs1121213; rs12134151; rs12534506; rs2135996; rs2477432; rs2980813; rs3768480; rs496590; rs8036481 | rs16845580; rs538628; rs35810066 |
|  | Fat | rs4741351; rs621893 | rs11130222; rs1121213; rs12134151; rs12534506; rs2135996; rs2477432; rs2980813; rs3768480; rs496590; rs8036481 | rs17552299 |
|  | Protein | rs4741351; rs621893 | rs11130222; rs1121213; rs12134151; rs12534506; rs2135996; rs2477432; rs2980813; rs3768480; rs496590; rs8036481 | rs1603981 |
| College | BMI | NA | rs2280405; rs238257; rs791903 | rs12134600; rs1225046; rs752579 |
|  | MDD | NA | rs10511431; rs16903920; rs2280405; rs238257; rs791903 | rs12134600; rs9289301 |
|  | Smoking | NA | rs10007915; rs16903920; rs2280405; rs238257; rs791903 | NA |
|  | Drinking | NA | rs10007915; rs16903920; rs2280405; rs238257; rs791903 | rs10445335; rs1487441; rs1225046; rs7729356 |
|  | Carbohydrate | rs10511431 | rs10007915; rs16903920; rs2280405; rs238257; rs791903 | rs10445335; rs2921930 |
|  | Fat | rs10511431 | rs10007915; rs16903920; rs2280405; rs238257; rs791903 | rs2921930 |
|  | Protein | rs10511431 | rs10007915; rs16903920; rs2280405; rs238257; rs791903 | rs2417349 |
| BMI | EC | rs184397922 | rs1558902; rs1979755; rs815610; rs4604474 | NA |
|  | BE | rs184397922; rs78338135 | rs1558902; rs1979755; rs4604474; rs815610 | NA |
|  | GERD | rs184397922; | rs1558902; rs1979755; rs815610; rs4604474 | NA |
| MDD | EC | NA | rs1214608; rs1152589; rs10759934 | rs9830950; rs28803900; rs71637418 |
|  | BE | rs184397922; rs78338135 | rs10759934; rs1152589; rs1214608 | NA |
|  | GERD | rs559710859; | rs1214608; rs1152589; rs10759934 | rs117473501; rs117849130; rs61533748 |
| Smoking | EC | NA | rs4357572; rs1193237; rs787362; rs4027747; rs9343820 | NA |
|  | BE | rs77785986; rs559710859; rs138795348; rs145406940 | rs1193237; rs4027747; rs4357572; rs787362; rs9343820 | NA |
|  | GERD | rs559710859; | rs4357572; rs1193237; rs787362; rs4027747; rs9343820 | rs75383625 |
| Drinking | EC | NA | rs2311766; rs35191471; rs1443641; rs4501371; rs11238438; rs28601761; rs9297173; rs1929431 | NA |
|  | BE | rs150820055; | rs11238438; rs1443641; rs1929431; rs35191471; rs4501371; rs9297173 | NA |
|  | GERD | rs150820055; | rs35191471; rs1443641; rs4501371; rs11238438; rs9297173; rs1929431 | NA |
| Carbohydrate | EC | NA | rs17580974; rs1104608; rs339844; rs1069267; rs904239 | NA |
|  | BE | NA | rs1069267; rs1104608; rs17580974; rs339844; rs904239 | NA |
|  | GERD | NA | rs17580974; rs1104608; rs339844; rs1069267; rs904239 | rs6725221; rs12636984 |
| Fat | EC | rs139930990; rs11549609; rs78765346 | rs12086856; rs873756 | NA |
|  | BE | rs139930990; rs78765346 | rs12086856; rs873756 | NA |
|  | GERD | rs78765346; rs139930990; rs11549609 | rs12086856; rs873756 | NA |
| Protein | EC | rs12098045; rs61806142; rs75681717 | rs2582908 | NA |
|  | BE | rs75681717; | rs2582908 | NA |
|  | GERD | rs75681717; rs12098045; rs61806142 | rs2582908 | NA |

## Table S5 The univariable MR analysis results of the causal effects of education traits on EC, BE, and GERD.

| Exposure | Outcome | Number of SNPs | F-statistic | Pleiotropy test* | | Heterogeneity test | | | | IVW method‡ | Odds ratio (95%CI) | *P-*value |
| --- | --- | --- | --- | --- | --- | --- | --- | --- | --- | --- | --- | --- |
|  |  |  |  | MR-Egger Intercept (95% CI) | *P*-value | Cochran’s Q-statistic | DF | I^2^, %† | *P* of Cochran’s Q-statistic |  |  |  |
| EduYears | EC | 364 | 24.99 | 0.002 (-0.019-0.024) | 8.44E-01 | 347.83 | 363 | 0.00 | 7.08E-01 | fixed-effect IVW | 0.64 (0.44-0.94) | 2.10E-02 |
|  | BE | 362 | 25.01 | 0.004 (-0.004-0.012) | 2.79E-01 | 331.85 | 361 | 0.00 | 8.62E-01 | fixed-effect IVW | 0.86 (0.75-0.99) | 3.81E-02 |
|  | GERD | 362 | 25.01 | -0.002 (-0.006-0.003) | 5.21E-01 | 457.71 | 361 | 21.13 | 4.12E-04 | fixed-effect IVW | 0.62 (0.58-0.67) | 2.62E-37 |
| College | EC | 113 | 20.75 | 0.022 (-0.032-0.076) | 4.29E-01 | 102.81 | 112 | 0.00 | 7.21E-01 | fixed-effect IVW | 0.97 (0.80-1.18) | 7.65E-01 |
|  | BE | 112 | 20.80 | -0.007 (-0.029-0.015) | 5.56E-01 | 137.86 | 111 | 19.48 | 4.28E-02 | fixed-effect IVW | 0.96 (0.90-1.03) | 3.04E-01 |
|  | GERD | 111 | 20.78 | -0.002 (-0.015-0.01) | 7.24E-01 | 152.25 | 110 | 27.75 | 4.77E-03 | random-effect IVW | 0.93 (0.89-0.97) | 9.67E-04 |

*: When there was significant pleiotropy (MR-Egger Intercept test P < 0.05), the MR-Egger model was used.

†: The formula for I^2^ is (Q-DF) /Q;

‡: When there was significant heterogeneity (I^2^ > 25% and P < 0.05), the random-effect IVW model was used, otherwise the fixed-effect IVW model was used.

## Table S6 The univariable MR analysis results of the causal effects of education traits on modifiable risk factors.

| Exposure | Outcome | Number of SNPs | F-statistic | Pleiotropy test* | | Heterogeneity test | | | | IVW method‡ | Odds ratio (95%CI) | *P*-value |
| --- | --- | --- | --- | --- | --- | --- | --- | --- | --- | --- | --- | --- |
|  |  |  |  | MR-Egger Intercept (95% CI) | *P*-value | Cochran’s Q-statistic | DF | I^2^, %† | *P* of Cochran’s Q-statistic |  |  |  |
| EduYears | BMI | 358 | 25.06 | -0.002 (-0.005-0) | 5.55E-02 | 781.76 | 357 | 54.33 | 8.37E-34 | random-effect IVW | 0.84 (0.80-0.88) | 1.61E-13 |
|  | MDD | 358 | 25.04 | -0.003 (-0.009-0.002) | 2.04E-01 | 623.43 | 357 | 42.74 | 9.09E-17 | random-effect IVW | 0.79 (0.73-0.87) | 3.20E-07 |
|  | Smoking | 359 | 25.07 | -0.002 (-0.005-0.002) | 3.40E-01 | 633.94 | 358 | 43.53 | 1.21E-17 | random-effect IVW | 0.76 (0.71-0.80) | 2.22E-19 |
|  | Drinking | 352 | 24.61 | 0.000 (-0.002-0.001) | 5.74E-01 | 725.95 | 351 | 51.65 | 2.59E-28 | random-effect IVW | 1.02 (0.99-1.04) | 1.01E-01 |
|  | Carbohydrate | 359 | 24.96 | -0.001 (-0.003-0.001) | 2.28E-01 | 502.01 | 358 | 28.69 | 7.27E-07 | random-effect IVW | 1.00 (0.97-1.03) | 9.05E-01 |
|  | Fat | 361 | 24.99 | 0.001 (-0.001-0.002) | 3.54E-01 | 532.12 | 360 | 32.35 | 8.93E-09 | random-effect IVW | 0.96 (0.94-0.99) | 7.99E-03 |
|  | Protein | 362 | 24.97 | -0.001 (-0.002-0.001) | 4.28E-01 | 492.14 | 361 | 26.65 | 5.07E-06 | random-effect IVW | 0.98 (0.95-1.01) | 1.21E-01 |
| College | BMI | 111 | 20.82 | -0.001 (-0.006-0.005) | 8.53E-01 | 153.08 | 110 | 28.14 | 4.18E-03 | random-effect IVW | 0.95 (0.93-0.97) | 3.59E-07 |
|  | MDD | 110 | 20.79 | -0.011 (-0.023-0) | 5.86E-02 | 150.25 | 109 | 27.45 | 5.43E-03 | random-effect IVW | 0.94 (0.90-0.98) | 4.49E-03 |
|  | Smoking | 112 | 20.72 | 0.002 (-0.006-0.01) | 6.34E-01 | 156.05 | 111 | 28.87 | 3.13E-03 | random-effect IVW | 0.93 (0.91-0.96) | 1.82E-06 |
|  | Drinking | 108 | 20.74 | 0.002 (-0.001-0.005) | 2.09E-01 | 207.36 | 107 | 48.40 | 2.13E-08 | random-effect IVW | 1.01 (1.00-1.02) | 7.92E-02 |
|  | Carbohydrate | 109 | 20.66 | -0.002 (-0.005-0.002) | 3.92E-01 | 130.03 | 108 | 16.94 | 7.32E-02 | fixed-effect IVW | 1.00 (0.99-1.01) | 8.30E-01 |
|  | Fat | 110 | 20.66 | 0 (-0.004-0.004) | 9.88E-01 | 123.44 | 109 | 11.70 | 1.63E-01 | fixed-effect IVW | 0.98 (0.97-0.99) | 3.62E-03 |
|  | Protein | 110 | 20.66 | 0.001 (-0.003-0.005) | 5.60E-01 | 168.12 | 109 | 35.16 | 2.41E-04 | random-effect IVW | 0.99 (0.97-1.01) | 8.32E-02 |

*: When there was significant pleiotropy (MR-Egger Intercept test P < 0.05), the MR-Egger model was used.

†: The formula for I^2^ is (Q-DF) /Q;

‡: When there was significant heterogeneity (I^2^ > 25% and P < 0.05), the random-effect IVW model was used, otherwise the fixed-effect IVW model was used.

## Table S7 The univariable MR analysis results of the causal effects of modifiable risk factors on EC, BE, and GERD.

| Exposure | Outcome | Number of SNPs | F-statistic | Pleiotropy test* | | Heterogeneity test | | | | IVW method‡ | Odds ratio (95%CI) | *P*-value |
| --- | --- | --- | --- | --- | --- | --- | --- | --- | --- | --- | --- | --- |
|  |  |  |  | MR-Egger Intercept (95% CI) | *P*-value | Cochran’s Q-statistic | DF | I^2^, %† | *P* of Cochran’s Q-statistic |  |  |  |
| BMI | EC | 228 | 32.86 | -0.012 (-0.033-0.008) | 2.44E-01 | 240.96 | 227 | 5.79 | 2.50E-01 | fixed-effect IVW | 1.51 (1.09-2.09) | 1.30E-02 |
|  | BE | 228 | 32.86 | 0.002 (-0.006-0.01) | 5.62E-01 | 259.07 | 227 | 12.38 | 7.07E-02 | fixed-effect IVW | 1.19 (1.06-1.33) | 4.28E-03 |
|  | GERD | 228 | 32.86 | 0.002 (-0.003-0.007) | 3.46E-01 | 382.46 | 227 | 40.65 | 4.80E-10 | random-effect IVW | 1.17 (1.08-1.27) | 9.68E-05 |
| MDD | EC | 200 | 20.52 | 0.015 (-0.001-0.031) | 6.21E-02 | 181.63 | 199 | 0.00 | 8.06E-01 | fixed-effect IVW | 1.06 (0.92-1.24) | 4.06E-01 |
|  | BE | 202 | 20.53 | 0.001 (-0.005-0.007) | 8.14E-01 | 208.82 | 201 | 3.74 | 3.38E-01 | fixed-effect IVW | 1.05 (1.00-1.11) | 5.60E-02 |
|  | GERD | 200 | 20.5 | 0.004 (0-0.008) | 6.01E-02 | 267.53 | 199 | 25.62 | 8.52E-04 | random-effect IVW | 1.12 (1.08-1.16) | 5.48E-10 |
| Smoking | EC | 205 | 30.33 | 0.002 (-0.016-0.019) | 8.58E-01 | 221.27 | 204 | 7.81 | 1.94E-01 | fixed-effect IVW | 1.25 (1.01-1.54) | 3.79E-02 |
|  | BE | 201 | 30.16 | 0.002 (-0.004-0.008) | 4.27E-01 | 196.24 | 200 | 0.00 | 5.62E-01 | fixed-effect IVW | 1.09 (1.01-1.17) | 3.40E-02 |
|  | GERD | 203 | 30.65 | 0.003 (0-0.007) | 5.47E-02 | 232.31 | 202 | 13.05 | 7.06E-02 | fixed-effect IVW | 1.08 (1.03-1.12) | 4.38E-04 |
| Drinking | EC | 266 | 28.63 | 0.001 (-0.015-0.016) | 9.18E-01 | 276.67 | 265 | 4.22 | 2.99E-01 | fixed-effect IVW | 1.36 (0.79-2.33) | 2.66E-01 |
|  | BE | 267 | 28.61 | -0.003 (-0.008-0.003) | 3.15E-01 | 276.44 | 266 | 3.78 | 3.17E-01 | fixed-effect IVW | 0.84 (0.69-1.02) | 7.85E-02 |
|  | GERD | 267 | 28.61 | -0.001 (-0.004-0.002) | 5.76E-01 | 382.49 | 266 | 30.46 | 3.62E-06 | random-effect IVW | 0.85 (0.75-0.96) | 1.04E-02 |
| Carbohydrate | EC | 157 | 16.56 | -0.003 (-0.031-0.025) | 8.17E-01 | 186.95 | 156 | 16.56 | 4.60E-02 | fixed-effect IVW | 1.67 (0.96-2.90) | 6.73E-02 |
|  | BE | 157 | 16.56 | -0.002 (-0.01-0.006) | 6.72E-01 | 141.51 | 156 | 0.00 | 7.91E-01 | fixed-effect IVW | 0.96 (0.79-1.16) | 6.57E-01 |
|  | GERD | 155 | 20.07 | -0.005 (-0.011-0) | 5.63E-02 | 195.62 | 154 | 21.28 | 1.31E-02 | fixed-effect IVW | 1.03 (0.92-1.14) | 6.13E-01 |
| Fat | EC | 133 | 24.49 | 0.008 (-0.017-0.033) | 5.36E-01 | 143.32 | 132 | 7.90 | 2.36E-01 | fixed-effect IVW | 0.71 (0.40-1.27) | 2.46E-01 |
|  | BE | 134 | 20.09 | -0.005 (-0.013-0.003) | 1.87E-01 | 113.28 | 133 | 0.00 | 8.91E-01 | fixed-effect IVW | 0.96 (0.78-1.18) | 7.08E-01 |
|  | GERD | 133 | 24.49 | 0.003 (-0.002-0.007) | 2.70E-01 | 139.41 | 132 | 5.32 | 3.12E-01 | fixed-effect IVW | 1.04 (0.93-1.16) | 4.81E-01 |
| Protein | EC | 129 | 18.51 | 0.007 (-0.021-0.035) | 6.30E-01 | 143.23 | 128 | 10.63 | 1.69E-01 | fixed-effect IVW | 0.60 (0.33-1.09) | 9.34E-02 |
|  | BE | 131 | 18.56 | -0.002 (-0.011-0.008) | 7.45E-01 | 124.57 | 130 | 0.00 | 6.18E-01 | fixed-effect IVW | 0.99 (0.80-1.23) | 9.21E-01 |
|  | GERD | 129 | 18.51 | 0.003 (-0.002-0.007) | 2.70E-01 | 154.02 | 128 | 16.89 | 5.84E-02 | fixed-effect IVW | 1.14 (1.01-1.27) | 2.81E-02 |

*: When there was significant pleiotropy (MR-Egger Intercept test P < 0.05), the MR-Egger model was used.

†: The formula for I^2^ is (Q-DF) /Q;

‡: When there was significant heterogeneity (I^2^ > 25% and P < 0.05), the random-effect IVW model was used, otherwise the fixed-effect IVW model was used.

## Table S8 The multivariable MR analysis results of the single causal effects of individual modifiable risk factors on EC, BE, and GERD after adjusting for education traits.

| Mediator | Adjustment factors | Outcome | Number of SNPs | Conditional F-statistics | Pleiotropy test* | | Heterogeneity test | | | | multivariable IVW | | multivariable MR Egger | | QHET method‡ | |  |
| --- | --- | --- | --- | --- | --- | --- | --- | --- | --- | --- | --- | --- | --- | --- | --- | --- | --- |
|  |  |  |  |  | MR-Egger Intercept (95% CI) | *P*-value | Cochran’s Q-statistic | DF | I^2^, %† | *P*-value | OR (95% CI) | *P*-value | OR (95% CI) | *P*-value | OR (95% CI) | *P*-value |  |
| BMI | EduYears | EC | 592 | 12.917 | -0.002(-0.011-0.006) | 5.70E-01 | 585.38 | 589 | 0.00 | 5.34E-01 | 1.50  (1.11-2.04) | 9.29E-03 | 1.53  (1.09-2.14) | 0.013 | - | - |  |
|  |  | BE | 590 | 12.947 | 0.001 (-0.002-0.004) | 4.97E-01 | 591.62 | 587 | 0.78 | 4.39E-01 | 1.14  (1.02-1.27) | 2.23E-02 | 1.13  (1.00-1.27) | 0.057 | - | - |  |
|  |  | GERD | 590 | 12.946 | -0.001(-0.003-0.001) | 3.22E-01 | 794.46 | 587 | 26.11 | 2.14E-08 | 1.16  (1.06-1.26) | 1.07E-03 | 1.11  (1.03-1.19) | 0.005 | 1.16  (1.06-1.26) | 0.001 |  |
| MDD | EduYears | EC | 564 | 8.311 | -0.008(-0.018-0.003) | 1.51E-01 | 525.93 | 561 | 0.00 | 8.53E-01 | 1.08  (0.94-1.24) | 2.71E-01 | 1.05  (0.91-1.21) | 0.516 | - | - |  |
|  |  | BE | 564 | 8.355 | 0.003 (-0.001-0.007) | 1.33E-01 | 540.28 | 561 | 0.00 | 7.28E-01 | 1.05  (1.00-1.10) | 5.70E-02 | 1.07  (1.01-1.13) | 0.012 | - | - |  |
|  |  | GERD | 562 | 8.285 | 0.000 (-0.002-0.002) | 9.59E-01 | 700.19 | 559 | 20.16 | 4.22E-05 | 1.11  (1.07-1.14) | 1.60E-09 | 1.12  (1.08-1.15) | 0.000 | - | - |  |
| Smoking | EduYears | EC | 569 | 10.727 | 0.002 (-0.007-0.012) | 6.15E-01 | 568.55 | 566 | 0.45 | 4.62E-01 | 1.25  (1.02-1.53) | 3.04E-02 | 1.27  (1.03-1.57) | 0.027 | - | - |  |
|  |  | BE | 563 | 10.688 | 0.001 (-0.002-0.005) | 5.52E-01 | 525.14 | 560 | 0.00 | 8.52E-01 | 1.09  (1.02-1.17) | 1.66E-02 | 1.09  (1.01-1.18) | 0.029 | - | - |  |
|  |  | GERD | 565 | 10.696 | 0.000 (-0.002-0.002) | 9.23E-01 | 652.64 | 562 | 13.89 | 4.80E-03 | 1.08  (1.03-1.13) | 5.03E-04 | 1.07  (1.03-1.12) | 0.002 | - | - |  |
| Drinking | EduYears | EC | 630 | 13.399 | -0.001(-0.009-0.007) | 8.28E-01 | 621.78 | 627 | 0.00 | 5.51E-01 | 1.44  (0.87-2.38) | 1.59E-01 | 1.34  (0.79-2.26) | 0.280 | - | - |  |
|  |  | BE | 629 | 13.465 | 0.001(-0.002-0.004) | 3.82E-01 | 607.91 | 626 | 0.00 | 6.91E-01 | 0.90  (0.75-1.08) | 2.45E-01 | 0.91  (0.75-1.1) | 0.307 | - | - |  |
|  |  | GERD | 629 | 13.414 | -0.001(-0.002-0.001) | 4.37E-01 | 792.53 | 626 | 21.01 | 6.43E-06 | 0.88  (0.79-0.98) | 1.93E-02 | 0.88  (0.79-0.99) | 0.033 | - | - |  |
| Carbohydrate | EduYears | EC | 521 | 7.340 | 0.009 (-0.001-0.02) | 8.90E-02 | 537.09 | 518 | 3.55 | 2.72E-01 | 1.30  (0.77-2.18) | 3.29E-01 | 1.15  (0.68-1.94) | 0.594 | - | - |  |
|  |  | BE | 519 | 7.368 | -0.001(-0.005-0.003) | 6.08E-01 | 470.96 | 516 | 0.00 | 9.23E-01 | 0.91  (0.77-1.08) | 2.65E-01 | 0.91  (0.76-1.09) | 0.307 | - | - |  |
|  |  | GERD | 519 | 7.368 | -0.001(-0.004-0.001) | 2.44E-01 | 637.79 | 516 | 19.10 | 1.94E-04 | 0.97  (0.87-1.09) | 6.32E-01 | 0.99  (0.88-1.10) | 0.791 | - | - |  |
| Fat | EduYears | EC | 497 | 6.795 | 0.004 (-0.008-0.015) | 5.14E-01 | 490.69 | 494 | 0.00 | 5.34E-01 | 0.91  (0.53-1.55) | 7.25E-01 | 0.89  (0.51-1.55) | 0.676 | - | - |  |
|  |  | BE | 496 | 6.826 | 0.004 (0.000-0.008) | 5.70E-02 | 446.08 | 493 | 0.00 | 9.36E-01 | 0.99  (0.82-1.18) | 8.82E-01 | 1.01  (0.83-1.23) | 0.906 | - | - |  |
|  |  | GERD | 495 | 6.816 | -0.001(-0.004-0.001) | 2.30E-01 | 574.85 | 492 | 14.41 | 5.75E-03 | 1.03  (0.92-1.15) | 5.92E-01 | 1.03  (0.92-1.16) | 0.626 | - | - |  |
| Protein | EduYears | EC | 493 | 6.475 | -0.001(-0.013-0.012) | 9.15E-01 | 489.49 | 490 | 0.00 | 4.98E-01 | 0.64  (0.37-1.11) | 1.16E-01 | 0.56  (0.31-1.00) | 0.051 | - | - |  |
|  |  | BE | 493 | 6.547 | 0.003 (-0.002-0.007) | 2.38E-01 | 454.32 | 490 | 0.00 | 8.74E-01 | 1.03  (0.85-1.25) | 7.27E-01 | 1.05  (0.84-1.3) | 0.679 | - | - |  |
|  |  | GERD | 491 | 6.437 | 0.000 (-0.003-0.002) | 8.75E-01 | 590.39 | 488 | 17.34 | 9.89E-04 | 1.15  (1.03-1.30) | 1.71E-02 | 1.15  (1.02-1.3) | 0.027 | - | - |  |
| BMI | College | EC | 341 | 19.623 | -0.003(-0.012-0.006) | 4.61E-01 | 343.83 | 338 | 1.70 | 4.02E-01 | 1.45  (1.04-2.01) | 2.69E-02 | 1.36  (0.97-1.91) | 0.079 | - | - |  |
|  |  | BE | 340 | 19.691 | 0.001 (-0.002-0.005) | 4.73E-01 | 397.39 | 337 | 15.20 | 1.30E-02 | 1.17  (1.03-1.33) | 1.72E-02 | 1.20  (1.06-1.37) | 0.006 | - | - |  |
|  |  | GERD | 339 | 19.749 | 0.001 (-0.001-0.003) | 5.28E-01 | 515.9 | 336 | 34.87 | 9.28E-10 | 1.19  (1.08-1.31) | 3.02E-04 | 1.14  (1.06-1.23) | 0.001 | 1.19  (1.08-1.31) | 0.000 |  |
| MDD | College | EC | 313 | 13.194 | -0.006(-0.017-0.005) | 3.06E-01 | 284.06 | 310 | 0.00 | 8.52E-01 | 1.05  (0.91-1.21) | 4.84E-01 | 1.04  (0.90-1.21) | 0.578 | - | - |  |
|  |  | BE | 314 | 13.317 | 0.001 (-0.003-0.006) | 5.51E-01 | 348.04 | 311 | 10.64 | 7.26E-02 | 1.04  (0.99-1.10) | 1.38E-01 | 1.05  (0.99-1.11) | 0.107 | - | - |  |
|  |  | GERD | 311 | 13.248 | 0.001 (-0.002-0.003) | 5.06E-01 | 405.47 | 308 | 24.04 | 1.58E-04 | 1.12  (1.08-1.16) | 1.95E-09 | 1.12  (1.08-1.16) | 0.000 | - | - |  |
| Smoking | College | EC | 318 | 18.360 | 0.006 (-0.009-0.022) | 4.22E-01 | 325.4 | 315 | 3.20 | 3.31E-01 | 1.29  (0.75-2.20) | 3.60E-01 | 1.31  (0.76-2.25) | 0.325 | - | - |  |
|  |  | BE | 313 | 18.329 | 0.000 (-0.004-0.003) | 8.32E-01 | 332.04 | 310 | 6.64 | 1.86E-01 | 1.09  (1.01-1.18) | 3.55E-02 | 1.09  (1.00-1.17) | 0.040 | - | - |  |
|  |  | GERD | 314 | 18.506 | 0.000 (-0.002-0.002) | 8.58E-01 | 375.87 | 311 | 17.26 | 6.84E-03 | 1.07  (1.03-1.12) | 2.40E-03 | 1.07  (1.03-1.12) | 0.002 | - | - |  |
| Drinking | College | EC | 379 | 19.751 | 0.001(-0.007-0.01) | 7.35E-01 | 379.78 | 376 | 1.00 | 4.36E-01 | 1.30  (0.76-2.21) | 3.39E-01 | 1.29  (0.76-2.21) | 0.345 | - | - |  |
|  |  | BE | 379 | 19.834 | -0.001(-0.004-0.002) | 5.65E-01 | 414.55 | 376 | 9.30 | 8.31E-02 | 0.86  (0.71-1.06) | 1.54E-01 | 0.85  (0.70-1.04) | 0.124 | - | - |  |
|  |  | GERD | 378 | 19.887 | -0.001(-0.003-0.001) | 2.19E-01 | 521.36 | 375 | 28.07 | 8.02E-07 | 0.81  (0.70-0.94) | 4.43E-03 | 0.87  (0.77-0.97) | 0.015 | 0.81  (0.70-0.94) | 0.004 |  |
| Carbohydrate | College | EC | 270 | 12.560 | 0.008(-0.004-0.019) | 2.10E-01 | 290.21 | 267 | 8.00 | 1.57E-01 | 1.57  (0.90-2.74) | 1.16E-01 | 1.48  (0.85-2.6) | 0.167 | - | - |  |
|  |  | BE | 269 | 12.641 | -0.003(-0.007-0.002) | 2.22E-01 | 279.35 | 266 | 4.78 | 2.75E-01 | 0.96  (0.79-1.16) | 6.86E-01 | 0.99  (0.81-1.19) | 0.881 | - | - |  |
|  |  | GERD | 268 | 12.684 | -0.002(-0.004-0.001) | 2.18E-01 | 359.23 | 265 | 26.23 | 1.01E-04 | 1.36  (1.05-1.75) | 1.92E-02 | 1.01  (0.90-1.14) | 0.848 | 1.36  (1.05-1.75) | 0.019 |  |
| Fat | College | EC | 246 | 11.632 | -0.008(-0.021-0.005) | 2.42E-01 | 245.71 | 243 | 1.10 | 4.39E-01 | 0.72  (0.40-1.27) | 2.57E-01 | 0.69  (0.38-1.23) | 0.203 | - | - |  |
|  |  | BE | 246 | 11.681 | 0.002 (-0.003-0.007) | 4.19E-01 | 251.71 | 243 | 3.46 | 3.37E-01 | 0.98  (0.80-1.20) | 8.17E-01 | 0.97  (0.79-1.18) | 0.738 | - | - |  |
|  |  | GERD | 244 | 11.726 | 0.000 (-0.002-0.003) | 7.43E-01 | 295.03 | 241 | 18.31 | 9.96E-03 | 1.03  (0.91-1.16) | 6.38E-01 | 1.03  (0.92-1.16) | 0.610 | - | - |  |
| Protein | College | EC | 242 | 11.889 | -0.012(-0.026-0.003) | 1.16E-01 | 247.31 | 239 | 3.36 | 3.42E-01 | 0.72  (0.40-1.30) | 2.77E-01 | 0.71  (0.4-1.29) | 0.261 | - | - |  |
|  |  | BE | 243 | 11.981 | 0.001 (-0.004-0.007) | 6.11E-01 | 260.51 | 240 | 7.87 | 1.73E-01 | 1.04  (0.84-1.30) | 7.07E-01 | 1.03  (0.83-1.28) | 0.804 | - | - |  |
|  |  | GERD | 240 | 11.974 | 0.000(-0.003-0.003) | 8.72E-01 | 305.47 | 237 | 22.41 | 1.78E-03 | 1.14  (1.01-1.29) | 4.03E-02 | 1.11  (0.99-1.26) | 0.077 | - | - |  |

*: When there was significant pleiotropy (MR-Egger Intercept test P < 0.05), the MR-Egger model was used.

†: The formula for I^2^ is (Q-DF) /Q.

‡: When there was significant heterogeneity (I^2^ > 25% and P < 0.05), the multivariable QHET result was the main results; otherwise, the multivariable IVW result was the main results.

## Table S9 The results of the mediation effect test that without statistically significant.

| **Exposure** | **Mediator** | **Outcome** | **Total Effect:** | **Direct Effect:** | **Direct Effect:** | **Mediation Effect** | ***P*-value** |
| --- | --- | --- | --- | --- | --- | --- | --- |
|  |  |  | **βc (95% CI) ^*^** | **βa (95% CI) ^†^** | **βb (95% CI) ^†^** | **(95% CI)** § |  |
| EduYears | MDD | EC | -0.444(-0.822, -0.067) | -0.232(-0.321, -0.143) | 0.076(-0.059, 0.212) | -0.018(-0.05, 0.015) | 0.282 |
| EduYears | Drinking | EC | -0.444(-0.822, -0.067) | 0.02(-0.004, 0.043) | 0.364(-0.142, 0.869) | 0.007(-0.006, 0.020) | 0.285 |
| EduYears | Carbohydrate | EC | -0.444(-0.822, -0.067) | 0.002(-0.026, 0.029) | 0.259(-0.26, 0.778) | 0.0004(-0.0067, 0.0076) | 0.906 |
| EduYears | Fat | EC | -0.444(-0.822, -0.067) | -0.038(-0.066, -0.01) | -0.096(-0.631, 0.439) | 0.004(-0.017, 0.024) | 0.727 |
| EduYears | Protein | EC | -0.444(-0.822, -0.067) | -0.023(-0.046, 0) | -0.443(-0.994, 0.108) | 0.01(-0.006, 0.027) | 0.219 |
| EduYears | MDD | BE | -0.145(-0.282, -0.008) | -0.232(-0.321, -0.143) | 0.048(-0.001, 0.098) | -0.011(-0.023, 0.001) | 0.074 |
| EduYears | Drinking | BE | -0.145(-0.282, -0.008) | 0.02(-0.004, 0.043) | -0.107(-0.288, 0.073) | -0.002(-0.006, 0.002) | 0.343 |
| EduYears | Carbohydrate | BE | -0.145(-0.282, -0.008) | 0.002(-0.026, 0.029) | -0.097(-0.267, 0.073) | -0.0002(-0.0028, 0.0025) | 0.905 |
| EduYears | Fat | BE | -0.145(-0.282, -0.008) | -0.038(-0.066, -0.01) | -0.014(-0.193, 0.166) | 0.001(-0.006, 0.007) | 0.882 |
| EduYears | Protein | BE | -0.145(-0.282, -0.008) | -0.023(-0.046, 0) | 0.034(-0.157, 0.226) | -0.001(-0.005, 0.004) | 0.731 |
| EduYears | Drinking | GERD | -0.473(-0.546, -0.4) | 0.02(-0.004, 0.043) | -0.132(-0.242, -0.022) | -0.003(-0.006, 0.001) | 0.179 |
| EduYears | Carbohydrate | GERD | -0.473(-0.546, -0.4) | 0.002(-0.026, 0.029) | -0.027(-0.136, 0.083) | 0.00001(-0.00016,0.00018) | 0.908 |
| EduYears | Fat | GERD | -0.473(-0.546, -0.4) | -0.038(-0.066, -0.01) | 0.031(-0.081, 0.143) | -0.001(-0.006, 0.003) | 0.600 |
| EduYears | Protein | GERD | -0.473(-0.546, -0.4) | -0.023(-0.046, 0) | 0.143(0.026, 0.26) | -0.003(-0.008, 0.001) | 0.128 |
| College | Drinking | GERD | -0.077(-0.115, -0.038) | 0.011(-0.001, 0.022) | -0.155(-0.271, -0.038) | -0.002(-0.004, 0.001) | 0.146 |
| College | Carbohydrate | GERD | -0.077(-0.115, -0.038) | 0.001(-0.011, 0.013) | 0.011(-0.104, 0.125) | 0.00001(-0.0002, 0.0002) | 0.908 |
| College | Fat | GERD | -0.077(-0.115, -0.038) | -0.018(-0.03, -0.006) | 0.03(-0.085, 0.144) | -0.001(-0.003, 0.002) | 0.617 |
| College | Protein | GERD | -0.077(-0.115, -0.038) | -0.015(-0.03, -0.001) | 0.125(0.005, 0.244) | -0.002(-0.004, 0.001) | 0.145 |

*Total Effect βc: the effect of educational attainment on the risk of EC, BE, and GERD. Only the results for which the total effect is statistically significant are shown here.

†Direct effect βa: the effect of educational attainment on the risk of modifiable risk factors.

‡Direct effect βb: the effect of modifiable risk factors on the risk of EC, BE, and GERD after adjusting two educational attainment traits.

§Mediation effect: $\beta_{a}\times\beta_{b}$; 95% CI of mediation effect: $\beta_{a}\times\beta_{b}\pm1.96\times\sqrt{\beta_{b}^{2}\times{se}_{a}^{2}+\beta_{a}^{2}\times{se}_{b}^{2}}$.

## Table S10 The multivariable MR analysis results of the combined causal effects of several modifiable risk factors on EC, BE, and GERD after adjusting for education traits.

| Mediator | Adjustment factors | Outcome | Number of SNPs | Conditional F-statistics | Pleiotropy test* | | Heterogeneity test | | | | multivariable IVW | | multivariable MR Egger | |  |
| --- | --- | --- | --- | --- | --- | --- | --- | --- | --- | --- | --- | --- | --- | --- | --- |
|  |  |  |  |  | MR-Egger Intercept (95% CI) | *P*-value | Cochran’s Q-statistic | DF | I^2^, %† | *P*-value | OR (95% CI) | *P*-value | OR (95% CI) | *P*-value |  |
| BMI | EduYears | EC | 797 | 9.927 | 0.000(-0.007,0.006) | 9.08E-01 | 774.65 | 793 | 0.00 | 0.673 | 1.43(1.06,1.93) | 2.06E-02 | 1.47(1.07,2.03) | 1.90E-02 |  |
| Smoking |  |  |  | 7.782 |  |  |  |  |  |  | 1.23(1.01,1.5) | 4.43E-02 | 1.23(1.00,1.51) | 4.60E-02 |  |
| BMI | EduYears | BE | 791 | 9.893 | 0.001(-0.002,0.003) | 5.11E-01 | 744.37 | 787 | 0.00 | 0.859 | 1.11(1,1.24) | 4.93E-02 | 1.09(0.97,1.23) | 1.33E-01 |  |
| Smoking |  |  |  | 7.734 |  |  |  |  |  |  | 1.08(1.01,1.16) | 2.77E-02 | 1.08(1.00,1.16) | 4.00E-02 |  |
| BMI | EduYears | GERD | 993 | 9.764 | 0.000(-0.001,0.001) | 7.21E-01 | 1199.77 | 988 | 17.65 | 0.00E+00 | 1.08(1.02,1.15) | 1.44E-02 | 1.1(1.03,1.17) | 5.00E-03 |  |
| MDD |  |  |  | 6.415 |  |  |  |  |  |  | 1.12(1.08,1.15) | 1.55E-12 | 1.12(1.09,1.16) | 0.00E+00 |  |
| Smoking |  |  |  | 7.786 |  |  |  |  |  |  | 1.05(1.01,1.1) | 1.27E-02 | 1.05(1.01,1.1) | 2.40E-02 |  |
| BMI | College | GERD | 742 | 9.681 | 0.001(0.000,0.002) | 1.99E-01 | 967.09 | 737 | 23.79 | 0.00E+00 | 1.12(1.04,1.19) | 1.38E-03 | 1.11(1.04,1.19) | 2.00E-03 |  |
| MDD |  |  |  | 6.463 |  |  |  |  |  |  | 1.13(1.09,1.17) | 1.19E-12 | 1.13(1.09,1.17) | 0.00E+00 |  |
| Smoking |  |  |  | 7.942 |  |  |  |  |  |  | 1.05(1.01,1.1) | 2.93E-02 | 1.05(1.01,1.1) | 2.50E-02 |  |

*: When there was significant pleiotropy (MR-Egger Intercept test P < 0.05), the MR-Egger model was used.

†: The formula for I^2^ is (Q-DF) /Q.

‡: When there was significant heterogeneity (I^2^ > 25% and P < 0.05), the multivariable QHET result was the main results; otherwise, the multivariable IVW result was the main results.

## Table S11 The complementary MR analysis results of the causal effects of education traits on EC, BE, and GERD.

| Exposure | Mediator | Number of SNPs | Maximum likelihood | | MR Egger | | IVW-SIMEXS (weighted) | | Weighted median | | Penalized weighted median | |  |  |  |  |  |  |  |  |  |  |
| --- | --- | --- | --- | --- | --- | --- | --- | --- | --- | --- | --- | --- | --- | --- | --- | --- | --- | --- | --- | --- | --- | --- |
|  |  |  | OR (95% CI) | *P*-value | OR (95% CI) | *P*-value | OR (95% CI) | *P*-value | OR (95% CI) | *P*-value | OR (95% CI) | *P*-value |  |  |  |  |  |  |  |  |  |  |
| EduYears | EC | 364 | 0.64 (0.44-0.95) | 2.46E-02 | 0.55 (0.12-2.47) | 4.39E-01 | 0.63 (0.43-0.93) | 1.92E-02 | 0.67 (0.38-1.18) | 1.63E-01 | 0.67 (0.39-1.16) | 1.55E-01 |  |  |  |  |  |  |  |  |  |  |
|  | BE | 362 | 0.87 (0.75-1) | 4.28E-02 | 0.65 (0.38-1.11) | 1.16E-01 | 0.86 (0.75-0.98) | 2.84E-02 | 0.78 (0.63-0.95) | 1.65E-02 | 0.77 (0.63-0.94) | 1.15E-02 |  |  |  |  |  |  |  |  |  |  |
|  | GERD | 362 | 0.61 (0.57-0.66) | 2.05E-37 | 0.69 (0.5-0.95) | 2.51E-02 | 0.61 (0.56-0.67) | <2e-16 | 0.62 (0.56-0.7) | 4.12E-16 | 0.62 (0.55-0.69) | 3.99E-18 |  |  |  |  |  |  |  |  |  |  |
| College | EC | 113 | 0.97 (0.79-1.19) | 7.63E-01 | 0.63 (0.21-1.87) | 4.06E-01 | 0.97 (0.79-1.18) | 7.56E-01 | 1.06 (0.8-1.4) | 7.05E-01 | 1.06 (0.8-1.39) | 7.04E-01 |  |  |  |  |  |  |  |  |  |  |
|  | BE | 112 | 0.96 (0.89-1.04) | 3.16E-01 | 1.1 (0.7-1.72) | 6.77E-01 | 0.96 (0.88-1.05) | 3.58E-01 | 1.02 (0.91-1.13) | 7.73E-01 | 1.02 (0.91-1.13) | 7.52E-01 |  |  |  |  |  |  |  |  |  |  |
|  | GERD | 111 | 0.93 (0.89-0.96) | 1.28E-04 | 0.97 (0.76-1.24) | 8.09E-01 | 0.92 (0.88-0.97) | 1.24E-03 | 0.94 (0.89-0.99) | 3.16E-02 | 0.94 (0.89-0.99) | 3.19E-02 |  | 3.70E-02 | 0.9411889 | 0.8905919 | 0.9946606 | 3.16E-02 | 0.9411853 | 0.8904965 | 0.9947594 | 3.19E-02 |

## Table S12 The complementary MR analysis results of the causal effects of education traits on modifiable risk factors.

| Exposure | Mediator | Number of SNPs | Maximum likelihood | | MR Egger | | IVW-SIMEXS (weighted) | | Weighted median | | Penalized weighted median | |
| --- | --- | --- | --- | --- | --- | --- | --- | --- | --- | --- | --- | --- |
|  |  |  | OR (95% CI) | *P*-value | OR (95% CI) | *P*-value | OR (95% CI) | *P*-value | OR (95% CI) | *P*-value | OR (95% CI) | *P*-value |
| EduYears | BMI | 358 | 0.84 (0.81-0.86) | 2.38E-26 | 0.97 (0.83-1.14) | 7.43E-01 | 0.84 (0.8-0.88) | 2.09E-12 | 0.85 (0.8-0.89) | 2.78E-10 | 0.84 (0.8-0.88) | 6.16E-11 |
|  | MDD | 358 | 0.78 (0.73-0.84) | 1.01E-11 | 0.99 (0.69-1.42) | 9.68E-01 | 0.79 (0.72-0.86) | 5.79E-07 | 0.87 (0.78-0.97) | 1.36E-02 | 0.87 (0.79-0.97) | 1.06E-02 |
|  | Smoking | 359 | 0.75 (0.71-0.78) | 9.94E-33 | 0.85 (0.66-1.08) | 1.78E-01 | 0.75 (0.7-0.8) | <2e-16 | 0.79 (0.73-0.85) | 1.06E-09 | 0.79 (0.73-0.85) | 2.85E-10 |
|  | Drinking | 352 | 1.02 (1-1.04) | 1.75E-02 | 1.05 (0.95-1.15) | 3.42E-01 | 1.02 (1-1.05) | 1.06E-01 | 1.01 (0.98-1.03) | 6.00E-01 | 1 (0.97-1.03) | 9.43E-01 |
|  | Carbohydrate | 359 | 1.07 (0.96-1.2) | 2.31E-01 | 0.99 (0.96-1.03) | 7.04E-01 | 1 (0.97-1.03) | 8.92E-01 | 0.99 (0.95-1.03) | 6.23E-01 | 0.95 (0.83-1.08) | 4.14E-01 |
|  | Fat | 361 | 0.96 (0.94-0.99) | 1.49E-03 | 0.91 (0.81-1.02) | 1.22E-01 | 0.96 (0.93-0.99) | 7.98E-03 | 0.96 (0.93-1) | 4.49E-02 | 0.97 (0.93-1) | 6.00E-02 |
|  | Protein | 361 | 0.98 (0.95-1) | 5.02E-02 | 1.02 (0.92-1.14) | 7.23E-01 | 0.98 (0.95-1) | 9.02E-02 | 0.97 (0.94-1.01) | 1.48E-01 | 0.96 (0.93-1) | 3.04E-02 |
| College | BMI | 111 | 0.95 (0.93-0.97) | 3.56E-09 | 0.96 (0.86-1.08) | 4.90E-01 | 0.95 (0.93-0.97) | 1.44E-06 | 0.97 (0.94-0.99) | 8.16E-03 | 0.97 (0.94-0.99) | 1.22E-02 |
|  | MDD | 110 | 0.94 (0.91-0.98) | 1.06E-03 | 1.18 (0.93-1.49) | 1.69E-01 | 0.94 (0.9-0.98) | 4.87E-03 | 0.97 (0.92-1.02) | 2.05E-01 | 0.97 (0.92-1.02) | 2.34E-01 |
|  | Smoking | 112 | 0.93 (0.91-0.95) | 2.23E-08 | 0.9 (0.77-1.05) | 1.90E-01 | 0.93 (0.9-0.96) | 5.48E-06 | 0.93 (0.9-0.96) | 6.52E-05 | 0.93 (0.89-0.96) | 4.62E-05 |
|  | Drinking | 108 | 1.01 (1-1.02) | 1.75E-02 | 0.97 (0.91-1.04) | 3.56E-01 | 1.01 (1-1.02) | 8.06E-02 | 1.01 (1-1.03) | 4.80E-02 | 1.01 (1-1.03) | 4.84E-02 |
|  | Carbohydrate | 109 | 1 (0.99-1.01) | 8.30E-01 | 1 (0.99-1.01) | 8.45E-01 | 1 (0.99-1.02) | 8.36E-01 | 1.03 (0.96-1.11) | 3.81E-01 | 1 (0.98-1.02) | 8.26E-01 |
|  | Fat | 110 | 0.98 (0.97-0.99) | 3.94E-03 | 0.98 (0.91-1.06) | 6.22E-01 | 0.98 (0.97-0.99) | 8.15E-03 | 0.98 (0.96-1) | 3.05E-02 | 0.98 (0.96-1) | 3.12E-02 |
|  | Protein | 110 | 0.99 (0.97-1) | 3.51E-02 | 0.96 (0.88-1.05) | 3.80E-01 | 0.99 (0.97-1) | 8.40E-02 | 0.99 (0.98-1.01) | 5.73E-01 | 0.99 (0.98-1.01) | 5.81E-01 |

## Table S13 The complementary MR analysis results of the causal effects of modifiable risk factors on EC, BE, and GERD.

| Exposure | Mediator | Number of SNPs | Maximum likelihood | | MR Egger | | IVW-SIMEXS (weighted) | | Weighted median | | Penalized weighted median | |
| --- | --- | --- | --- | --- | --- | --- | --- | --- | --- | --- | --- | --- |
|  |  |  | OR (95% CI) | *P*-value | OR (95% CI) | *P*-value | OR (95% CI) | *P*-value | OR (95% CI) | *P*-value | OR (95% CI) | *P*-value |
| BMI | EC | 228 | 1.53 (1.1-2.13) | 1.14E-02 | 2.66 (0.97-7.31) | 5.85E-02 | 1.52 (1.08-2.15) | 1.77E-02 | 1.43 (0.86-2.37) | 1.66E-01 | 1.43 (0.85-2.39) | 1.74E-01 |
|  | BE | 228 | 1.19 (1.05-1.34) | 4.70E-03 | 1.07 (0.73-1.56) | 7.38E-01 | 1.19 (1.05-1.36) | 8.03E-03 | 1.14 (0.95-1.37) | 1.49E-01 | 1.16 (0.97-1.4) | 9.94E-02 |
|  | GERD | 228 | 1.18 (1.11-1.26) | 4.19E-07 | 1.05 (0.82-1.34) | 6.90E-01 | 1.18 (1.08-1.28) | 1.47E-04 | 1.16 (1.05-1.29) | 3.71E-03 | 1.18 (1.06-1.3) | 2.50E-03 |
| MDD | EC | 200 | 1.07 (0.92-1.24) | 4.07E-01 | 0.86 (0.65-1.12) | 2.67E-01 | 1.07 (0.92-1.24) | 3.77E-01 | 0.96 (0.73-1.25) | 7.55E-01 | 0.96 (0.72-1.26) | 7.50E-01 |
|  | BE | 202 | 1.05 (1-1.11) | 5.70E-02 | 1.04 (0.94-1.15) | 4.09E-01 | 1.06 (1-1.12) | 6.20E-02 | 1.03 (0.93-1.14) | 5.37E-01 | 1.03 (0.93-1.14) | 5.38E-01 |
|  | GERD | 200 | 1.12 (1.09-1.16) | 2.25E-12 | 1.05 (0.97-1.13) | 2.52E-01 | 1.13 (1.08-1.17) | 4.17E-09 | 1.06 (1.01-1.11) | 2.09E-02 | 1.05 (1-1.1) | 4.42E-02 |
| Smoking | EC | 205 | 1.25 (1.01-1.55) | 4.05E-02 | 1.2 (0.76-1.9) | 4.27E-01 | 1.26 (1-1.58) | 4.80E-02 | 1.5 (1.03-2.19) | 3.43E-02 | 1.5 (1-2.27) | 5.13E-02 |
|  | BE | 201 | 1.09 (1.01-1.17) | 3.53E-02 | 1.03 (0.88-1.2) | 7.51E-01 | 1.09 (1.01-1.18) | 3.53E-02 | 1.17 (1.02-1.35) | 2.67E-02 | 1.17 (1.02-1.35) | 2.26E-02 |
|  | GERD | 203 | 1.08 (1.03-1.12) | 4.34E-04 | 0.99 (0.91-1.09) | 9.00E-01 | 1.08 (1.03-1.13) | 1.21E-03 | 1.04 (0.95-1.14) | 3.57E-01 | 1.04 (0.96-1.14) | 3.53E-01 |
| Drinking | EC | 266 | 1.37 (0.79-2.37) | 2.64E-01 | 1.28 (0.37-4.47) | 6.99E-01 | 1.37 (0.78-2.43) | 2.78E-01 | 1.5 (0.55-4.12) | 4.34E-01 | 1.49 (0.53-4.19) | 4.47E-01 |
|  | BE | 267 | 0.84 (0.69-1.02) | 7.93E-02 | 1.03 (0.66-1.63) | 8.84E-01 | 0.84 (0.68-1.03) | 8.82E-02 | 0.83 (0.56-1.22) | 3.42E-01 | 0.84 (0.56-1.25) | 3.98E-01 |
|  | GERD | 267 | 0.85 (0.77-0.95) | 2.77E-03 | 0.91 (0.69-1.21) | 5.35E-01 | 0.85 (0.74-0.96) | 1.12E-02 | 0.86 (0.68-1.08) | 1.98E-01 | 0.86 (0.68-1.07) | 1.83E-01 |
| Carbohydrate | EC | 157 | 1.7 (0.96-3) | 6.66E-02 | 2.01 (0.37-10.83) | 4.16E-01 | 1.71 (0.91-3.22) | 9.90E-02 | 2.33 (1.03-5.3) | 4.30E-02 | 2.37 (1.03-5.44) | 4.22E-02 |
|  | BE | 157 | 0.96 (0.79-1.16) | 6.61E-01 | 1.05 (0.67-1.64) | 8.44E-01 | 0.96 (0.79-1.16) | 6.39E-01 | 1.08 (0.79-1.49) | 6.28E-01 | 1.08 (0.79-1.49) | 6.25E-01 |
|  | GERD | 155 | 1.03 (0.92-1.15) | 6.11E-01 | 1.39 (1-1.93) | 5.21E-02 | 1.03 (0.91-1.17) | 6.37E-01 | 1.01 (0.86-1.18) | 9.06E-01 | 1.02 (0.87-1.2) | 8.04E-01 |
| Fat | EC | 133 | 0.71 (0.39-1.29) | 2.59E-01 | 0.48 (0.12-1.88) | 2.95E-01 | 0.7 (0.37-1.32) | 2.73E-01 | 0.78 (0.32-1.9) | 5.87E-01 | 0.77 (0.32-1.85) | 5.62E-01 |
|  | BE | 134 | 0.96 (0.78-1.18) | 7.09E-01 | 1.23 (0.81-1.87) | 3.31E-01 | 0.96 (0.79-1.17) | 7.07E-01 | 1.12 (0.83-1.52) | 4.58E-01 | 1.12 (0.82-1.54) | 4.71E-01 |
|  | GERD | 133 | 1.04 (0.93-1.17) | 4.82E-01 | 0.91 (0.71-1.18) | 4.95E-01 | 1.05 (0.93-1.18) | 4.66E-01 | 1.06 (0.89-1.26) | 5.10E-01 | 1.06 (0.89-1.26) | 4.91E-01 |
| Protein | EC | 129 | 0.59 (0.32-1.09) | 9.43E-02 | 0.44 (0.11-1.81) | 2.57E-01 | 0.59 (0.3-1.13) | 1.15E-01 | 0.92 (0.38-2.22) | 8.58E-01 | 0.94 (0.38-2.35) | 9.01E-01 |
|  | BE | 131 | 0.99 (0.79-1.23) | 9.20E-01 | 1.06 (0.66-1.71) | 8.06E-01 | 0.99 (0.79-1.23) | 9.09E-01 | 0.94 (0.68-1.3) | 6.92E-01 | 0.93 (0.69-1.27) | 6.70E-01 |
|  | GERD | 129 | 1.14 (1.01-1.28) | 3.17E-02 | 1.14 (0.86-1.51) | 3.60E-01 | 1.14 (1-1.3) | 5.03E-02 | 1.07 (0.9-1.27) | 4.47E-01 | 1.07 (0.9-1.28) | 4.46E-01 |

## Table S14 Verification results of potentially weak instrumental bias in causal effects of education traits on EC, BE, and GERD.

| Exposure | Outcome | Number of SNPs | Radial IVW | |
| --- | --- | --- | --- | --- |
|  |  |  | OR (95% CI) | *P*-value |
| EduYears | EC | 364 | 0.64 (0.44-0.93) | 1.84E-02 |
|  | BE | 362 | 0.86 (0.76-0.99) | 3.06E-02 |
|  | GERD | 362 | 0.62 (0.57-0.68) | 8.38E-30 |
| College | EC | 113 | 0.97 (0.8-1.17) | 7.55E-01 |
|  | BE | 112 | 0.96 (0.89-1.04) | 3.56E-01 |
|  | GERD | 111 | 0.93 (0.89-0.97) | 9.62E-04 |

## Table S15 Verification results of potentially weak instrumental bias in causal effects of education traits on modifiable risk factors.

| Exposure | Outcome | Number of SNPs | Radial IVW | |
| --- | --- | --- | --- | --- |
|  |  |  | OR (95% CI) | *P*-value |
| EduYears | BMI | 358 | 0.84 (0.8-0.88) | 1.54E-13 |
|  | MDD | 358 | 0.79 (0.73-0.87) | 3.21E-07 |
|  | Smoking | 359 | 0.75 (0.71-0.8) | 2.00E-19 |
|  | Drinking | 352 | 1.02 (1-1.04) | 1.01E-01 |
|  | Carbohydrate | 359 | 0.96 (0.94-0.99) | 1.49E-03 |
|  | Fat | 361 | 0.96 (0.94-0.99) | 7.98E-03 |
|  | Protein | 361 | 0.98 (0.95-1) | 8.77E-02 |
| College completion | BMI | 111 | 0.95 (0.93-0.97) | 3.67E-07 |
|  | MDD | 110 | 0.94 (0.9-0.98) | 4.47E-03 |
|  | Smoking | 112 | 0.93 (0.91-0.96) | 1.81E-06 |
|  | Drinking | 107 | 1.01 (1-1.02) | 7.93E-02 |
|  | Carbohydrate | 110 | 1 (0.99-1.01) | 8.45E-01 |
|  | Fat | 111 | 0.98 (0.97-0.99) | 6.26E-03 |
|  | Protein | 109 | 0.99 (0.97-1) | 8.32E-02 |

## Table S16 Verification results of potentially weak instrumental bias in causal effects of modifiable risk factors on EC, BE, and GERD.

| Exposure | Outcome | Number of SNPs | Radial IVW | |
| --- | --- | --- | --- | --- |
|  |  |  | OR (95% CI) | *P*-value |
| BMI | EC | 228 | 1.51 (1.08-2.11) | 1.59E-02 |
|  | BE | 228 | 1.19 (1.05-1.35) | 7.50E-03 |
|  | GERD | 228 | 1.17 (1.08-1.27) | 9.67E-05 |
| MDD | EC | 200 | 1.06 (0.92-1.23) | 3.84E-01 |
|  | BE | 202 | 1.05 (1.00-1.11) | 6.09E-02 |
|  | GERD | 200 | 1.12 (1.08-1.16) | 4.69E-10 |
| Smoking | EC | 205 | 1.25 (1.00-1.55) | 4.63E-02 |
|  | BE | 201 | 1.09 (1.01-1.17) | 3.24E-02 |
|  | GERD | 203 | 1.08 (1.03-1.12) | 1.04E-03 |
| Drinking | EC | 266 | 1.36 (0.78-2.36) | 2.76E-01 |
|  | BE | 267 | 0.84 (0.69-1.02) | 8.43E-02 |
|  | GERD | 267 | 0.85 (0.75-0.96) | 1.04E-02 |
| Carbohydrate | EC | 157 | 1.67 (0.91-3.06) | 9.47E-02 |
|  | BE | 157 | 0.96 (0.8-1.15) | 6.41E-01 |
|  | GERD | 155 | 1.03 (0.91-1.16) | 6.54E-01 |
| Fat | EC | 133 | 0.71 (0.39-1.3) | 2.66E-01 |
|  | BE | 134 | 0.96 (0.8-1.16) | 6.85E-01 |
|  | GERD | 133 | 1.04 (0.93-1.17) | 4.93E-01 |
| Protein | EC | 129 | 0.6 (0.32-1.13) | 1.13E-01 |
|  | BE | 131 | 0.99 (0.80-1.22) | 9.19E-01 |
|  | GERD | 129 | 1.14 (1.00-1.29) | 4.53E-02 |

## Table S17 MR analysis results of the effect of instrumental variables of education traits on EC, BE, and GERD screened by strict threshold criteria (P < 5e-8).

| Exposure* | Mediator | N | MR-Egger intercept test | Heterogeneity test‡ | | IVW method‡ | OR (95% CI) | *P*-value |
| --- | --- | --- | --- | --- | --- | --- | --- | --- |
|  |  |  |  | I2, %† | Q_*P* |  |  |  |
| EduYears | EC | 67 | 0.595 | 0.00 | 5.28E-01 | fixed-effect IVW | 0.53 (0.27-1.05) | 6.92E-02 |
|  | BE | 67 | 0.877 | 19.46 | 8.91E-02 | fixed-effect IVW | 0.91 (0.71-1.16) | 4.46E-01 |
|  | GERD | 67 | 0.383 | 30.01 | 1.27E-02 | random-effect IVW | 0.57 (0.49-0.67) | 3.45E-12 |
| College | EC | 3 | 0.894 | 0.00 | 6.69E-01 | fixed-effect IVW | 0.89 (0.38-2.09) | 7.82E-01 |
|  | BE | 3 | 0.571 | 71.87 | 2.86E-02 | random-effect IVW | 0.99 (0.55-1.78) | 9.72E-01 |
|  | GERD | 3 | 0.604 | 47.32 | 1.50E-01 | fixed-effect IVW | 0.84 (0.67-1.05) | 1.25E-01 |

†: The formula for I^2^ is (Q-DF) /Q;

‡: When there was significant heterogeneity (I^2^ > 25% and P < 0.05), the random-effect IVW model was used, otherwise the fixed-effect IVW model was used.

## Table S18 MR analysis results of the effect of instrumental variables of education traits on modifiable risk factors screened by strict threshold criteria (P < 5e-8).

| Exposure* | Mediator | N | MR-Egger intercept test | Heterogeneity test‡ | | IVW method‡ | OR (95% CI) | *P*-value |
| --- | --- | --- | --- | --- | --- | --- | --- | --- |
|  |  |  |  | I2, %† | Q_*P*† |  |  |  |
| EduYears | BMI | 67 | 0.069 | 72.66 | 7.98E-22 | random-effect IVW | 0.79 (0.71-0.88) | 2.64E-05 |
|  | MDD | 66 | 0.908 | 57.84 | 3.30E-09 | random-effect IVW | 0.85 (0.7-1.02) | 8.35E-02 |
|  | Smoking | 67 | 0.334 | 58.87 | 7.72E-10 | random-effect IVW | 0.71 (0.63-0.81) | 2.95E-07 |
|  | Drinking | 59 | 0.951 | 52.38 | 1.98E-06 | random-effect IVW | 1.03 (0.99-1.08) | 1.51E-01 |
|  | Carbohydrate | 64 | 0.090 | 50.40 | 3.23E-06 | random-effect IVW | 1.04 (0.98-1.1) | 2.02E-01 |
|  | Fat | 66 | 0.046 | 50.53 | 2.13E-06 | random-effect IVW | 0.92 (0.86-0.97) | 4.76E-03 |
|  | Protein | 66 | 0.123 | 54.97 | 5.86E-08 | random-effect IVW | 0.98 (0.92-1.04) | 5.62E-01 |
| College | BMI | 3 | 0.399 | 70.93 | 3.21E-02 | random-effect IVW | 0.9 (0.79-1.03) | 1.17E-01 |
|  | MDD | 3 | 0.437 | 43.53 | 1.70E-01 | fixed-effect IVW | 0.92 (0.76-1.12) | 4.22E-01 |
|  | Smoking | 3 | 0.572 | 25.11 | 2.63E-01 | fixed-effect IVW | 0.84 (0.74-0.94) | 4.01E-03 |
|  | Drinking | 3 | 0.397 | 0.00 | 3.73E-01 | fixed-effect IVW | 0.97 (0.94-1.01) | 1.03E-01 |
|  | Carbohydrate | 3 | 0.533 | 0.00 | 6.41E-01 | fixed-effect IVW | 1.08 (1.04-1.11) | 2.63E-05 |
|  | Fat | 3 | 0.533 | 0.00 | 7.80E-01 | fixed-effect IVW | 0.96 (0.92-1.02) | 1.64E-01 |
|  | Protein | 3 | 0.489 | 81.40 | 4.63E-03 | random-effect IVW | 0.97 (0.86-1.09) | 5.72E-01 |

†: The formula for I^2^ is (Q-DF) /Q;

‡: When there was significant heterogeneity (I^2^ > 25% and P < 0.05), the random-effect IVW model was used, otherwise the fixed-effect IVW model was used.

## Table S19 MR analysis results of the effect of instrumental variables of modifiable risk factors on EC, BE, and GERD screened by strict threshold criteria (P < 5e-8).

| Exposure* | Mediator | N | MR-Egger intercept test | Heterogeneity test‡ | | IVW method‡ | OR (95% CI) | *P*-value |
| --- | --- | --- | --- | --- | --- | --- | --- | --- |
|  |  |  |  | I2, %† | Q_*P* |  |  |  |
| BMI | EC | 66 | 0.629 | 0.00 | 8.87E-01 | fixed-effect IVW | 1.98 (1.25-3.13) | 3.74E-03 |
|  | BE | 66 | 0.814 | 0.00 | 5.19E-01 | fixed-effect IVW | 1.2 (1.01-1.41) | 3.46E-02 |
|  | GERD | 66 | 0.792 | 44.97 | 6.25E-05 | random-effect IVW | 1.12 (1.02-1.22) | 1.26E-02 |
| MDD | EC | 5 | 0.387 | 0.00 | 8.44E-01 | fixed-effect IVW | 0.97 (0.43-2.20) | 9.45E-01 |
|  | BE | 5 | 0.74 | 13.60 | 3.27E-01 | fixed-effect IVW | 1.16 (0.86-1.56) | 3.19E-01 |
|  | GERD | 5 | 0.151 | 36.80 | 1.76E-01 | fixed-effect IVW | 1.33 (1.14-1.56) | 3.21E-04 |
| Smoking | EC | 22 | 0.397 | 0.00 | 4.60E-01 | fixed-effect IVW | 1.11 (0.79-1.55) | 5.62E-01 |
|  | BE | 22 | 0.742 | 0.00 | 5.07E-01 | fixed-effect IVW | 1.13 (0.99-1.27) | 6.03E-02 |
|  | GERD | 22 | 0.13 | 0.00 | 6.68E-01 | fixed-effect IVW | 1.06 (0.99-1.13) | 7.57E-02 |
| Drinking | EC | 36 | 0.233 | 3.58 | 4.08E-01 | fixed-effect IVW | 1.07 (0.43-2.63) | 8.91E-01 |
|  | BE | 37 | 0.077 | 0.00 | 7.21E-01 | fixed-effect IVW | 1.08 (0.78-1.50) | 6.35E-01 |
|  | GERD | 37 | 0.749 | 44.60 | 2.17E-03 | random-effect IVW | 0.94 (0.75-1.19) | 6.09E-01 |
| Carbohydrate | EC | 11 | 0.195 | 0.00 | 4.80E-01 | fixed-effect IVW | 0.70 (0.15-3.29) | 6.54E-01 |
|  | BE | 11 | 0.488 | 0.00 | 5.97E-01 | fixed-effect IVW | 1.20 (0.69-2.09) | 5.26E-01 |
|  | GERD | 11 | 0.197 | 60.74 | 4.52E-03 | random-effect IVW | 0.86 (0.54-1.38) | 5.37E-01 |
| Fat | EC | 5 | 0.357 | 0.00 | 7.18E-01 | fixed-effect IVW | 0.56 (0.10-3.21) | 5.16E-01 |
|  | BE | 5 | 0.52 | 25.28 | 2.53E-01 | fixed-effect IVW | 1.23 (0.66-2.32) | 5.15E-01 |
|  | GERD | 5 | 0.81 | 0.00 | 4.10E-01 | fixed-effect IVW | 1.07 (0.77-1.50) | 6.74E-01 |
| Protein | EC | 7 | 0.981 | 0.00 | 9.70E-01 | fixed-effect IVW | 1.08 (0.21-5.57) | 9.25E-01 |
|  | BE | 7 | 0.669 | 2.64 | 4.05E-01 | fixed-effect IVW | 0.81 (0.45-1.47) | 4.95E-01 |
|  | GERD | 7 | 0.719 | 31.26 | 1.89E-01 | fixed-effect IVW | 1.01 (0.74-1.38) | 9.67E-01 |

†: The formula for I^2^ is (Q-DF) /Q.

‡: When there was significant heterogeneity (I^2^ > 25% and P < 0.05), the random-effect IVW model was used, otherwise the fixed-effect IVW model was used.

## Table S20 MR analysis results of the effect of instrumental variables of education traits on EC, BE, and GERD (using GWAS including UK Biobank individuals, Lee et al.) on EC, BE, and GERD.

| Exposure | Outcome | Number of SNPs | Pleiotropy test* | |  | Heterogeneity test‡ | | | | IVW method‡ | Odds ratio (95%CI) | *P-*value |
| --- | --- | --- | --- | --- | --- | --- | --- | --- | --- | --- | --- | --- |
|  |  |  | MR-Egger Intercept (95% CI) | *P*-value |  | Cochran’s Q-statistic | DF | I^2^, %† | *P* of Cochran’s Q-statistic |  |  |  |
| EduYears | EC | 1215 | 0.001(-0.010,0.011) | 8.92E-01 |  | 1223.67 | 1214 | 0.79 | 4.17E-01 | fixed-effect IVW | 0.76(0.58-0.99) | 4.66E-02 |
|  | BE | 1207 | 0.000(-0.001,0.001) | 7.45E-01 |  | 1146.67 | 1206 | 0.00 | 8.88E-01 | fixed-effect IVW | 0.88(0.80-0.97) | 8.05E-03 |
|  | GERD | 1216 | 0.000(-0.002,0.002) | 9.31E-01 |  | 1484.47 | 1215 | 18.15 | 1.52E-07 | fixed-effect IVW | 0.51(0.48-0.53) | 1.71E-124 |

*: When there was significant pleiotropy (MR-Egger Intercept test P < 0.05), the MR-Egger model was used.

†: The formula for I^2^ is (Q-DF) /Q.

‡: When there was significant heterogeneity (I^2^ > 25% and P < 0.05), the random-effect IVW model was used, otherwise the fixed-effect IVW model was used.

## Table S21 MR analysis results of the effect of instrumental variables of education traits on modifiable risk factors (using GWAS including UK Biobank individuals, Lee et al.) on modifiable risk factors.

| Exposure | Outcome | Number of SNPs | Pleiotropy test* | |  | Heterogeneity test‡ | | | | IVW method‡ | Odds ratio (95%CI) | *P*-value |
| --- | --- | --- | --- | --- | --- | --- | --- | --- | --- | --- | --- | --- |
|  |  |  | MR-Egger Intercept (95% CI) | *P*-value |  | Cochran’s Q-statistic | DF | I^2^, %† | *P* of Cochran’s Q-statistic |  |  |  |
| EduYears | BMI | 880 | 0.000(-0.001,0.002) | 7.82E-01 |  | 1865.46 | 879 | 52.82 | 4.44E-73 | random-effect IVW | 0.78(0.75,0.81) | 4.31E-40 |
|  | MDD | 1216 | 0.001(-0.001,0.004) | 3.04E-01 |  | 2180.37 | 1215 | 44.28 | 9.05E-58 | random-effect IVW | 0.71(0.67,0.76) | 9.27E-26 |
|  | Smoking | 1200 | -0.002(-0.004,0.000) | 3.50E-02 |  | 2437.91 | 1199 | 50.82 | 8.61E-87 | MR-Egger | 0.82(0.7,0.97) | 1.83E-02 |
|  | Drinking | 1200 | 0.000(-0.001,0.000) | 5.04E-01 |  | 3040.15 | 1199 | 60.56 | 2.94E-160 | random-effect IVW | 1.05(1.03,1.07) | 3.31E-07 |
|  | Carbohydrate | 1201 | 0.000(-0.001,0.001) | 6.97E-01 |  | 1890.56 | 1200 | 36.52 | 8.72E-34 | random-effect IVW | 1.01(0.99,1.04) | 1.82E-01 |
|  | Fat | 1201 | 0.000(0.000,0.001) | 2.07E-01 |  | 1711.33 | 1200 | 29.88 | 1.08E-20 | random-effect IVW | 0.93(0.91,0.95) | 4.47E-14 |
|  | Protein | 1201 | 0.000(-0.001,0.000) | 4.36E-01 |  | 1586.61 | 1200 | 24.37 | 3.29E-13 | fixed-effect IVW | 0.97(0.95,0.99) | 1.79E-03 |

*: When there was significant pleiotropy (MR-Egger test P < 0.05), the MR-Egger model was used.

†: The formula for I^2^ is (Q-DF) /Q.

‡: When there was significant heterogeneity (I^2^ > 25% and P < 0.05), the random-effect IVW model was used, otherwise the fixed-effect IVW model was used.

1. Okbay A;Beauchamp JP;Fontana MA, et al. Genome-wide association study identifies 74 loci associated with educational attainment. *Nature* 2016; 533: 539-42.

2. Rietveld CA;Medland SE;Derringer J, et al. GWAS of 126,559 individuals identifies genetic variants associated with educational attainment. *Science (New York, NY)* 2013; 340: 1467-71.

3. Locke AE;Kahali B;Berndt SI, et al. Genetic studies of body mass index yield new insights for obesity biology. *Nature* 2015; 518: 197-206.

4. Wray NR;Ripke S;Mattheisen M, et al. Genome-wide association analyses identify 44 risk variants and refine the genetic architecture of major depression. *Nat Genet* 2018; 50: 668-81.

5. Liu M;Jiang Y;Wedow R, et al. Association studies of up to 1.2 million individuals yield new insights into the genetic etiology of tobacco and alcohol use. *Nat Genet* 2019; 51: 237-44.

6. Meddens SFW;de Vlaming R;Bowers P, et al. Genomic analysis of diet composition finds novel loci and associations with health and lifestyle. *Mol Psychiatry* 2021; 26: 2056-69.

7. Sudlow C;Gallacher J;Allen N, et al. UK biobank: an open access resource for identifying the causes of a wide range of complex diseases of middle and old age. *PLoS Med* 2015; 12: e1001779.
